# Combinatorial *Gli* activity directs immune infiltration and tumor growth in pancreatic cancer

**DOI:** 10.1371/journal.pgen.1010315

**Published:** 2022-07-22

**Authors:** Michael K. Scales, Ashley Velez-Delgado, Nina G. Steele, Hannah E. Schrader, Anna M. Stabnick, Wei Yan, Nayanna M. Mercado Soto, Zeribe C. Nwosu, Craig Johnson, Yaqing Zhang, Daniel J. Salas-Escabillas, Rosa E. Menjivar, H. Carlo Maurer, Howard C. Crawford, Filip Bednar, Kenneth P. Olive, Marina Pasca di Magliano, Benjamin L. Allen

**Affiliations:** 1 Department of Cell and Developmental Biology, University of Michigan, Ann Arbor, Michigan, United States of America; 2 Department of Surgery, University of Michigan, Ann Arbor, Michigan, United States of America; 3 Department of Molecular and Integrative Physiology, University of Michigan, Ann Arbor, Michigan, United States of America; 4 Rogel Cancer Center, University of Michigan, Ann Arbor, Michigan, United States of America; 5 Cancer Biology Program, University of Michigan, Ann Arbor, Michigan, United States of America; 6 Cellular and Molecular Biology Program, University of Michigan, Ann Arbor, Michigan, United States of America; 7 Department of Medicine, Vagelos College of Physicians and Surgeons, Columbia University Irving Medical Center, New York city, New York, United States of America; 8 Internal Medicine II, School of Medicine, Technische Universität München, Munich, Germany; 9 Department of Surgery, Henry Ford Health System, Detroit, Michigan, United States of America; 10 Herbert Irving Comprehensive Cancer Center, Columbia University Irving Medical Center, New York city, New York, United States of America; Brigham and Women’s Hospital, UNITED STATES

## Abstract

Proper Hedgehog (HH) signaling is essential for embryonic development, while aberrant HH signaling drives pediatric and adult cancers. HH signaling is frequently dysregulated in pancreatic cancer, yet its role remains controversial, with both tumor-promoting and tumor-restraining functions reported. Notably, the GLI family of HH transcription factors (GLI1, GLI2, GLI3), remain largely unexplored in pancreatic cancer. We therefore investigated the individual and combined contributions of GLI1-3 to pancreatic cancer progression. At pre-cancerous stages, fibroblast-specific *Gli2/Gli3* deletion decreases immunosuppressive macrophage infiltration and promotes T cell infiltration. Strikingly, combined loss of *Gli1/Gli2/Gli3* promotes macrophage infiltration, indicating that subtle changes in *Gli* expression differentially regulate immune infiltration. In invasive tumors, *Gli2/Gli3* KO fibroblasts exclude immunosuppressive myeloid cells and suppress tumor growth by recruiting natural killer cells. Finally, we demonstrate that fibroblasts directly regulate macrophage and T cell migration through the expression of *Gli-*dependent cytokines. Thus, the coordinated activity of GLI1-3 directs the fibroinflammatory response throughout pancreatic cancer progression.

## Author summary

Throughout life, proper organ function relies on tightly regulated communication between diverse cell types. Disruptions to this intercellular communication can lead to disease, including pediatric and adult cancers. In pancreatic cancer, abnormal communication between tumor cells, fibroblasts and immune cells prevents the immune system from effectively recognizing and destroying malignant cells. Finding a way to correct this deficiency could create new therapeutic opportunities for this deadly disease. In this study, we investigated the role of Hedgehog signaling in fibroblasts, a central cell type in the pancreatic tumor microenvironment. Specifically, we explored the contribution of the GLI family of transcription factors (which mediate the cellular responses to Hedgehog signaling) to pancreatic cancer progression. We found that *Gli* genes are expressed in pancreatic fibroblasts and that the proteins encoded by these genes impact pancreatic tumor growth and directly control immune cell migration. This work identifies GLI transcription factors as key regulators of fibroblast-immune cell communication in pancreatic cancer.

## Introduction

Pancreatic ductal adenocarcinoma (PDA) remains a deadly malignancy, with a 5-year survival rate of 11% [1]. One contributing factor to this low survival rate is a lack of effective therapies. Although the mechanisms driving resistance to treatment are complex, one major barrier is the tumor microenvironment (TME). The TME of PDA is extremely heterogeneous, involving a complex network of endothelial cells, nerves, fibroblasts, and immune cells [2]. Within this network, fibroblasts function as critical nodes for intercellular signaling. Pancreatic fibroblasts contribute to pancreatic disease through a variety of means, including the production of extracellular matrix (ECM) and the secretion of pro-tumorigenic factors [3–7]. Fibroblasts also provide metabolic support [8–10], confer chemoresistance [7,11,12], facilitate immunosuppression [13,14], and restrict tumor perfusion via ECM deposition [15–17]. However, fibroblasts also have tumor-restricting roles [18–21]. These seemingly disparate functions could be explained by the observation that cancer-associated fibroblasts are heterogeneous [22–24], with different populations having different, potentially opposing functions. However, the mechanisms and signals used by fibroblast populations to affect disease progression are not fully understood.

Work from our lab and others has identified aberrant HH signaling as a feature of PDA [25–27]. In the context of PDA, HH ligands (primarily sonic hedgehog [SHH] and indian hedgehog [IHH]) are secreted by tumor cells and bind to the canonical receptor patched 1 (PTCH1) on fibroblasts [28–30]. Following ligand binding, the repressive activity of PTCH1 is inhibited, leading to the activation of smoothened (SMO), which in turn modulates the GLI family of HH transcription factors [31]. Although this paracrine model of HH signaling in pancreatic cancer is well-established, the contribution of HH signaling to pancreatic cancer progression remains controversial.

Previous mouse studies indicated that HH pathway inhibition improves chemotherapy delivery and extends survival [15]. However, clinical trials employing SMO inhibitors provided no clinical benefit or, in some cases, led to worse outcomes [32–34]. Further, genetic loss of *Shh* shortens survival in mouse models of PDA, suggesting that HH signaling has tumor-restraining roles [19,20]. One explanation for these contradictory results is that the level of HH pathway activity influences pancreatic tumor growth. Combinatorial targeting of HH pathway co-receptors revealed that partial reduction of HH signaling in fibroblasts promotes tumor growth, while near complete ablation of the stromal HH response fails to promote tumorigenesis [18]. More recent work demonstrated that pharmacologic HH pathway inhibition alters cancer-associated fibroblast (CAF) composition and immune infiltration in PDA, indicating that HH signaling impacts multiple cell types within the pancreatic TME [35]. However, the downstream consequences of HH pathway activity on pancreatic tumor growth, specifically the transcriptional outcomes of HH signal transduction in the pancreatic stroma, remain unexplored.

The GLI family of proteins (GLI1, GLI2, GLI3) are the transcriptional effectors of the HH pathway. GLI1 exclusively functions as a transcriptional activator, while GLI2 and GLI3 contain both activator and repressor domains [36]. In multiple tissues, GLI2 primarily acts as a transcriptional activator [37], while GLI3 functions as a transcriptional repressor [38]. Prior work from our group has demonstrated that GLI1 supports pancreatic tissue recovery following induction of acute pancreatitis or oncogenic *Kras*-driven injury [39]. However, the expression and function of GLI2 and GLI3 in PDA remain largely unknown. Further, the combined role of multiple GLIs during PDA progression has not been explored.

In this study, we investigated the role of GLI1-3 in PDA progression. We have determined that *Gli1*, *Gli2*, and *Gli3* are expressed in the healthy pancreas, and expand throughout PDA progression. At pre-cancerous stages, genetic deletion of *Gli2* and *Gli3* in fibroblasts reduces collagen deposition and dramatically alters immune infiltration. Specifically, stromal depletion of *Gli2* and *Gli3* leads to a decrease in immunosuppressive macrophage infiltration and an increase in T cells. However, deleting all three *Glis* in fibroblasts leads to an increase in macrophage infiltration and the exclusion of T cells. Further, mice lacking *Gli1*, *Gli2*, and *Gli3* display a widespread loss of pancreatic tissue, suggesting that a baseline level of GLI activity is necessary to maintain tissue integrity during disease progression. In invasive tumors, we have determined that the loss of *Gli2* and *Gli3* in fibroblasts decreases myeloid-derived suppressor cells (MDSCs) and increases natural killer (NK) cells, which in turn antagonize tumor growth. In contrast, *Gli1/Gli2/Gli3* KO fibroblasts recruit MDSCs and exclude NK cells, leading to sustained tumor growth. Together, our data demonstrate that the activities of all three GLIs regulate immune infiltration throughout PDA progression, and these GLI-driven changes determine tumor growth.

## Results

### *Gli1-3* are expressed in the pancreatic stroma and expand during PDA progression

**Fig 1:**
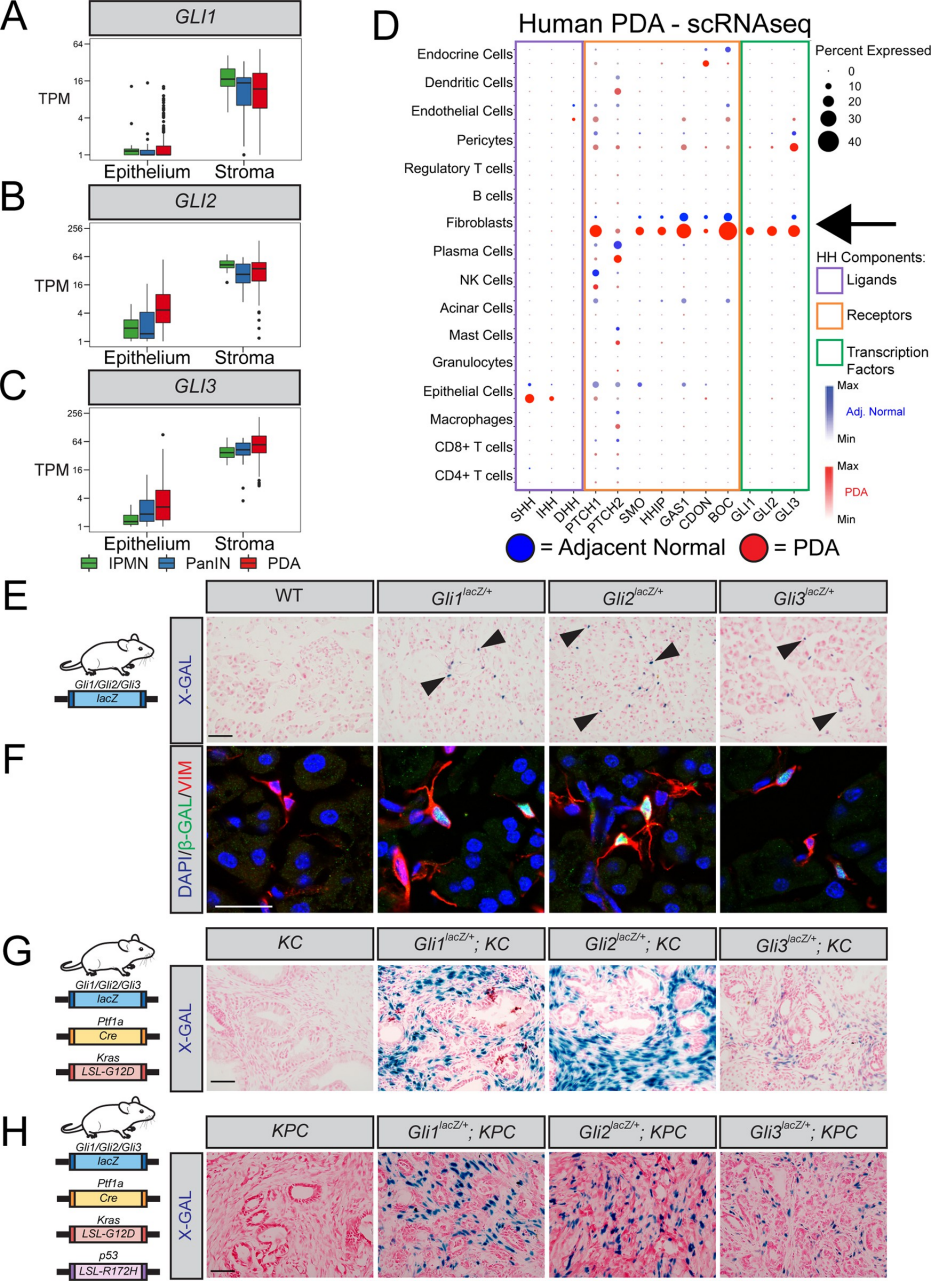
*Gli1-3* are expressed in the pancreatic stroma and expand during PDA progression. (**A–C**). Epithelial vs. Stromal *GLI* expression in human IPMN tissue (green, n = 19 Epithelial, n = 12 Stromal), PanIN (blue, n = 26 Epithelial, n = 23 Stromal), and PDA (red, n = 197 Epithelial, n = 124 Stromal), determined by LCM-RNAseq [40]. (**D**) Expression of HH pathway components in human PDA (red, n = 16) and adjacent normal pancreas (blue, n = 3), determined by scRNAseq [42]. Dot size indicates frequency. Dot color intensity indicates expression level. Boxes outline ligands (purple), receptors (orange), and transcription factors (green). Arrow indicates *GLI* expression in fibroblasts. (**E–H**) Expression analysis of healthy (**E-F**), PanIN (**G**), and PDA (**H**) pancreata from *Gli*^*lacZ*^ reporter mice (n ≥ 3 for all genotypes). X-gal staining (**E, G, H**) in blue. Arrowheads indicate *lacZ+* cells. Scale bar = 50μm. For immunofluorescent antibody analysis of healthy pancreata (**F**), antibodies detect β-Galactosidase (β-GAL, green) and fibroblasts (VIM, Red). DAPI (blue) denotes nuclei. Scale bar = 20 μm. Mouse drawing acquired from the open source repository SciDraw.io (doi.org/10.5281/zenodo.3925901).

To determine *GLI1-3* expression in human PDA, we analyzed epithelial and stromal samples isolated from PDA patients by laser-capture microdissection [40]. *GLI1-3* are predominantly expressed in the stroma (Fig 1A–1C), while HH ligands are expressed in the epithelium (S1A Fig). HH receptors are also enriched in the stroma (S2B and S2C Fig), consistent with the paracrine manner of HH signaling in PDA [25,26,28,30,41]. Further, all three *GLI*s are expressed by multiple types of pre-cancerous lesions, including both pancreatic intraepithelial neoplasia (PanIN) and intraductal papillary mucinous neoplasms (IPMNs). Since the stroma consists of diverse cell types, we analyzed a recently published single-cell RNA sequencing dataset [42] to precisely determine which cellular compartments express *GLI1-3*. Gene expression analysis revealed that fibroblasts are the primary source of *GLI1-3* in PDA (Fig 1D).

*Kras*^*LSL-G12D/+*^*;Ptf1a*^*Cre/+*^ (KC) [43] and *Kras*^*LSL-G12D/+*^*; p53*^*LSL-R172H/+*^*;Ptf1a*^*Cre/+*^ (KPC) [44] mouse models of PDA provide an opportunity to investigate disease progression in a system that faithfully recapitulates human disease. In both models, *Cre* expression by pancreatic progenitor cells activates oncogenic *Kras* [43,44]. Induction of acute pancreatitis (via caeruelin injections) synergizes with oncogenic *Kras* to produce widespread PanIN lesions in KC mice [45]. In KPC mice, CRE also drives the expression of a mutant *p53* allele, leading to the spontaneous development of invasive pancreatic tumors [44]. To determine if *Gli1-3* expression in mouse models is consistent with human disease, we utilized *Gli-lacZ* reporter mice [37,46,47]. X-gal staining (Fig 1E) and immunofluorescent (IF) detection of Beta Galactosidase (β-GAL; Figs 1F and S1D and S1E) in healthy mouse pancreata revealed that *Gli1*, *Gli2*, and *Gli3* are all expressed by fibroblasts surrounding blood vessels and pancreatic ducts. Further, *Gli2*- and *Gli3*-expressing fibroblasts also surround acinar cell clusters (Fig 1E and 1F). *Gli-lacZ* reporter mice were then crossed into KC and KPC models of PDA. X-gal staining of *Gli1-lacZ;KC*, *Gli2-lacZ;KC*, and *Gli3-lacZ*;*KC* tissue revealed that all three *Glis* expand within the stroma at PanIN stages (Fig 1G), and immunofluorescent detection of β-GAL indicated that *Gli* expression remains restricted to pancreatic fibroblasts (S1F–S1I Fig). Further, *Gli1-3* are expressed broadly within the tumor stroma of *Gli-lacZ; KPC* mice (Figs 1H and S1J). Together, these data demonstrate that the patterns of *Gli* expression observed in mouse models are consistent with human PDA.

### Conditional *Gli2* and *Gli3* deletion *in vivo* restricts immunosuppressive macrophages and promotes T cell infiltration

**Fig 2:**
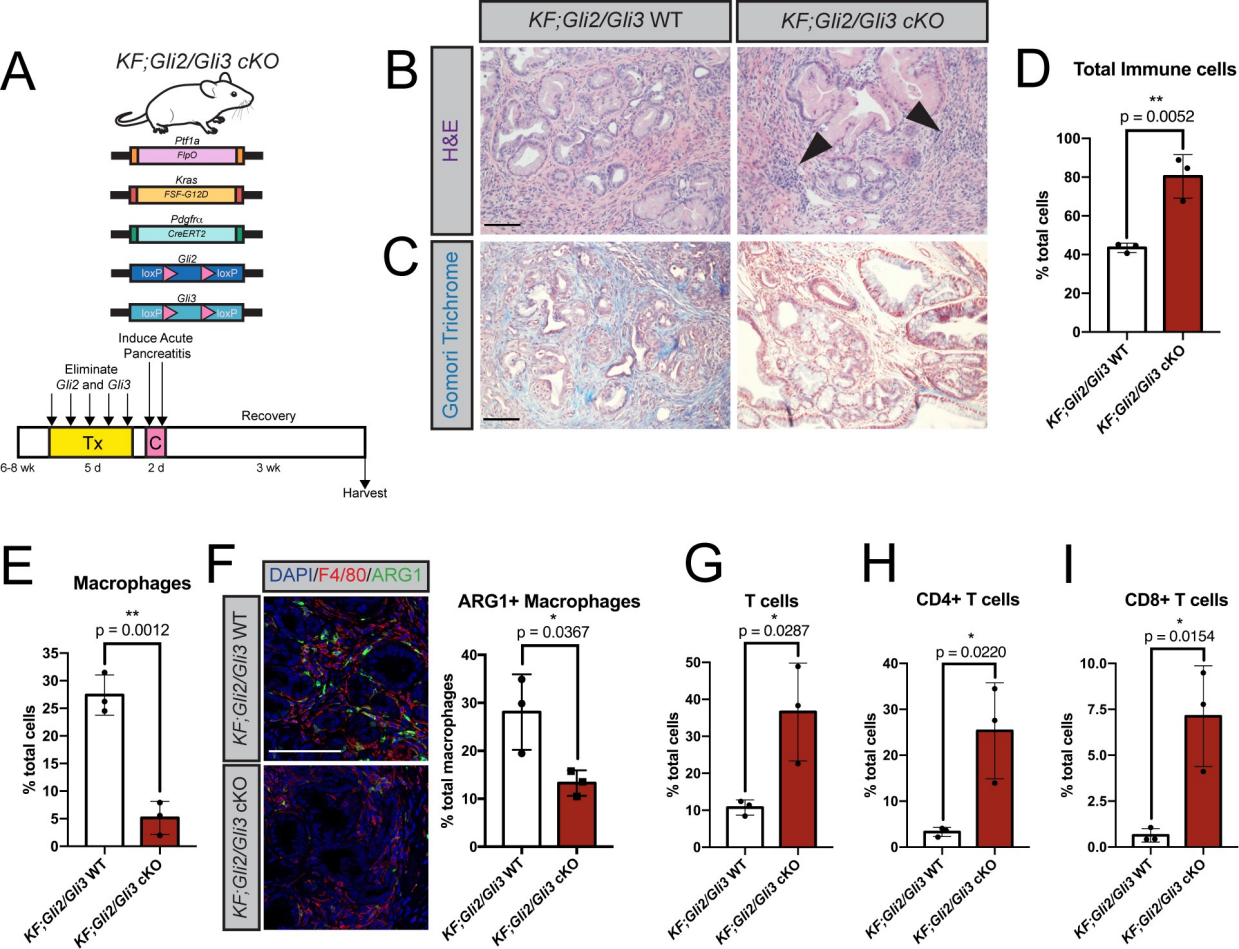
Conditional *Gli2* and *Gli3* deletion *in vivo* restricts immunosuppressive macrophages and promotes T cell infiltration. **A**) Cartoon depicting experimental strategy. Adult *KF; Pdgfrα*^*CreERt2/+*^*; Gli2*^*fl/fl*^*; Gli3*^*fl/fl*^ (*KF;Gli2/Gli3* cKO) mice were given tamoxifen (Tx, 200mg/kg) once a day for 5 days. Mice were then given 8 hourly injections of caerulein (C) over 2 days to induce pancreatitis. Pancreata were harvested 3 weeks later. **B-C**) H&E (**B**) and Gomori trichrome (**C**) staining of *KF;Gli2/Gli3* cKO mice (right) and *KF; Gli2*^*fl/fl*^*; Gli3*^*fl/fl*^ (*KF;Gli2/Gli3* WT) mice (left). Arrowheads indicate dense pockets of stromal cells. **D-E**) Flow cytometry analysis of total immune cells (**D**) and macrophages (**E**) in *KF;Gli2/Gli3* WT and *KF;Gli2/Gli3* cKO mice. **F**) Immunofluorescent antibody detection (left) and quantitation (right) of macrophages (F4/80, red) expressing arginase 1 (ARG1, green). DAPI staining in blue. **G-I**) Flow cytometry analysis of total T cells (**G**), CD4+ T cells (**H**), and CD8+ T Cells (**I**). N ≥ 3 for all genotypes. All P-values were determined by un-paired t-test. Scale bars = 100μm. Mouse drawing acquired from the open source repository SciDraw.io (doi.org/10.5281/zenodo.3925901).

The broad, stromal expression of *Gli1-3* throughout disease progression raised the question of *Gli* function in PDA. While *Gli1* has established roles in tissue recovery [39] and PanIN progression [48], the roles of stromal *Gli2* and *Gli3* during PDA progression are unknown. To target *Gli2* and *Gli3* in fibroblasts *in vivo*, we crossed the *Pdgfrα*^*CreER-T2*^ allele [49] into *Kras*^*FSF-G12D/+*^*;Ptf1a*^*FlpO/+*^ (KF) [50] mice. In this combined *KF;Pdgfrα*^*CreER-T2*^ model (Fig 2A), *Ptf1a*^*FlpO/+*^ drives oncogenic *Kras* expression and PanIN formation in the pancreatic epithelium, allowing us to utilize a CreER to inducibly delete *Gli* in pancreatic fibroblasts. Adult *KF;Pdgfrα*^*CreER-T2*^ mice were given tamoxifen daily for 5 days to induce recombination, followed by two days of caerulein injections to drive acute pancreatitis and initiate PanIN formation [51]. Mice were then harvested 3 weeks later once PanIN lesions established throughout the pancreas. Lineage tracing revealed that the *Pdgfrα*^*CreER-T2*^ allele effectively labels the neoplastic stroma (S2A Fig), and reduces *Gli2* and *Gli3* expression by approximately 70% each in *KF; Pdgfrα*^*CreER-T2*^*; Gli2*^*fl/fl*^; *Gli3*^*fl/fl*^ (*KF;Gli2/Gli3* cKO) mice (S2B Fig). Histologic analysis of *KF;Gli2/Gli3* cKO mice revealed a disrupted stroma, including an increased number of stromal cells and a decrease in collagen deposition compared to *KF; Gli2*^*fl/fl*^; *Gli3*^*fl/fl*^ (*KF;Gli2/Gli3* WT) mice (Fig 2B and 2C). However, initial characterization of fibroblasts from *KF;Gli2/Gli3* cKO mice revealed no inherent differences in proliferation compared to *KF;Gli2/Gli3* WT mice (S2C Fig). To determine which stromal cell types change as a result of *Gli2/Gli3* deletion, we performed flow cytometry analysis on *KF;Gli2/Gli3* cKO and *KF;Gli2/Gli3* WT tissue. Interestingly, we found that total immune cells increase following loss of *Gli2* and *Gli3*, yet total myeloid cells decrease (Figs 2D and S2D and S2E). When we examined different subpopulations of immune cells (Figs 2E–2I and S2D and S2F–S2J), we found a significant reduction in macrophages in *KF;Gli2/Gli3* cKO mice (Fig 2E). To assess which types of macrophages are impacted by the loss of *Gli2/Gli3*, we evaluated the expression of arginase 1 (ARG1), a marker of immunosuppressive tumor-associated macrophages (TAMs). Co-staining with ARG1 and the general macrophage marker F4/80 revealed a decrease in TAMs following the loss of *Gli2* and *Gli3* (Fig 2F). In contrast to this decrease in immunosuppressive TAMs, we found an increase in total T cells in *KF;Gli2/Gli3* cKO mice (Figs 2G and S2D). Analysis of different T cell populations revealed that both CD4+ and CD8+ T cells increased following the loss of *Gli2* and *Gli3* (Figs 2H and 2I, and S2D and S2H–S2J). Together, these data suggest that fibroblast-specific *Gli2*/*Gli3* deletion disrupts the immunosuppressive microenvironment in pancreatic neoplasia.

**Fig 3:**
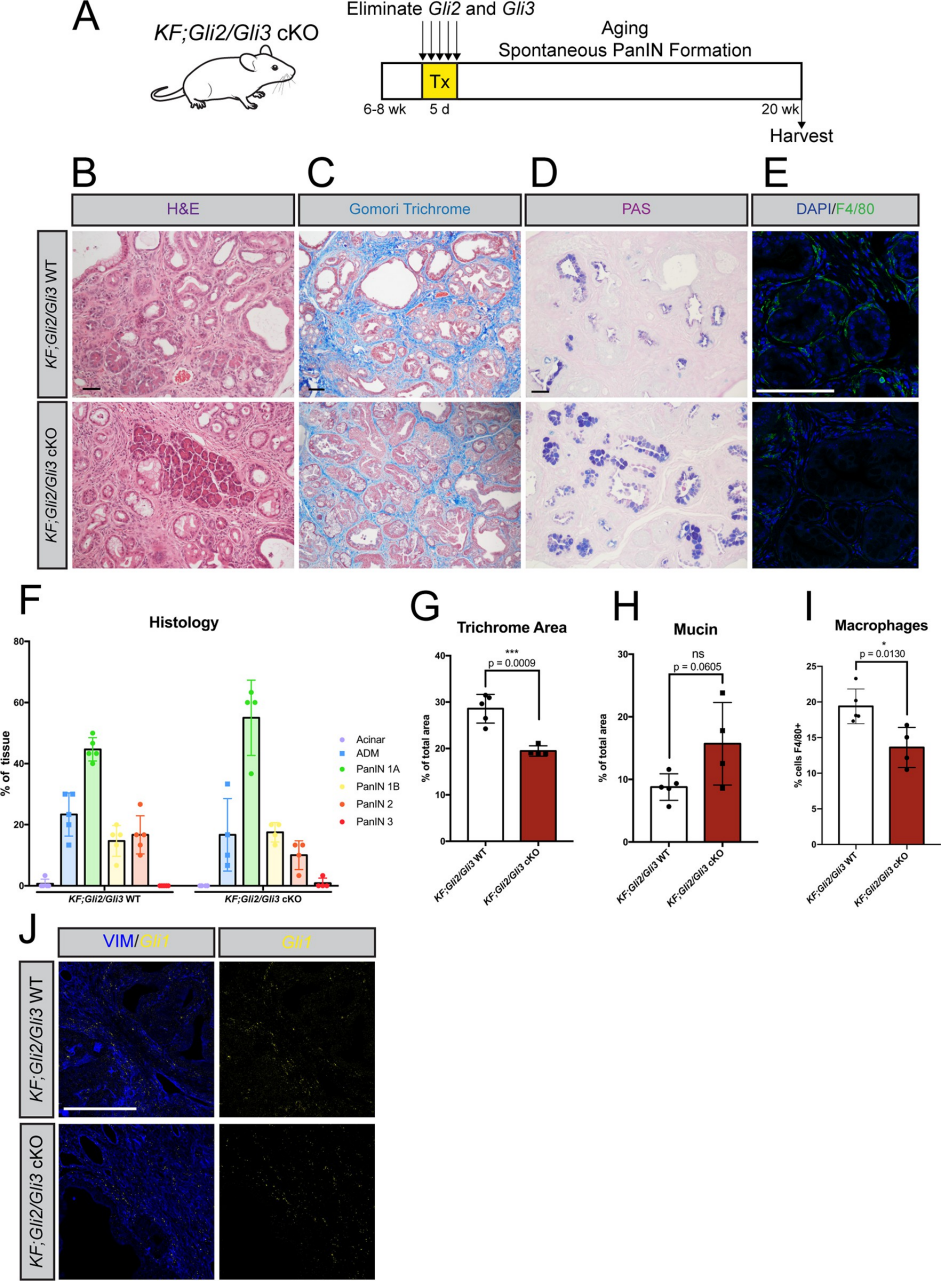
Loss of stromal *Gli2* and *Gli3* reduces collagen deposition and macrophage infiltration in a spontaneous PanIN model. **A**) Cartoon depicting experimental strategy. Adult *KF;Gli2/Gli3* cKO mice were given tamoxifen (Tx, 200 mg/kg) once a day for 5 days. Pancreata were harvested at 20 weeks of age. **B-D**) Histological analysis of *KF;Gli2/Gli3* cKO;*LSL-tdTomato/+* (bottom) pancreata compared to *KF;Gli2/Gli3* WT;*LSL-tdTomato/+* tissue (top), including H&E (**B**), Gomori Trichrome (**C**), and PAS (**D**) staining. **E**) Immunofluorescent antibody detection of macrophages (F4/80, green). DAPI staining in blue. **F**) Pathology analysis of PanIN progression in *KF;Gli2/Gli3* WT;*LSL-tdTomato/+* (left) and *KF;Gli2/Gli3* cKO;*LSL-tdTomato/+* (right) mice. **G-I**) Quantitation of collagen deposition (**G**), mucin (**H**), and macrophages (**I**) in *KF;Gli2/Gli3* WT;*LSL-tdTomato/+* and *KF;Gli2/Gli3* cKO;*LSL-tdTomato/+* mice. **J**) *Gli1* expression (yellow) in *KF;Gli2/Gli3* WT (top) and *KF;Gli2/Gli3* cKO mice (bottom), determined by RNAscope. Vimentin (VIM) staining in blue. Scale bar = 50 μm. N ≥ 3 for all genotypes. P-values were determined by un-paired t-test. Mouse drawing acquired from the open source repository SciDraw.io (doi.org/10.5281/zenodo.3925901).

Since immune suppression plays a crucial role in PDA [14], we investigated whether loss of *Gli2/Gli3* affects PanIN progression. However, caerulein administration in mutant *Kras* mice can synchronize and accelerate PanIN lesions [45]. We therefore utilized a spontaneous model in which *KF;Gli2/Gli3* cKO mice were aged to 20 weeks, when spontaneous PanIN formation is expected (Fig 3A and 3B). *KF;Gli2/Gli3* cKO mice present with reduced collagen deposition (Fig 3C and 3G) and a reduction in macrophage infiltration (Fig 3E and 3I). We also observed a trend toward increased cytoplasmic mucin, a feature of low-grade PanIN [52], in a majority of *KF;Gli2/Gli3* cKO mice (Fig 3D and 3H). However, histopathological analysis of the PanIN lesions indicated no difference in PanIN progression between *KF;Gli2/Gli3* cKO and *KF;Gli2/Gli3* WT mice (Fig 3F). Thus, while loss of *Gli2* and *Gli3* in the fibroblasts significantly impairs ECM accumulation and reduces the relative proportion of macrophages, it is not sufficient to alter PanIN progression.

### Combined *Gli1-3* deletion drives widespread tissue loss during PanIN progression

**Fig 4:**
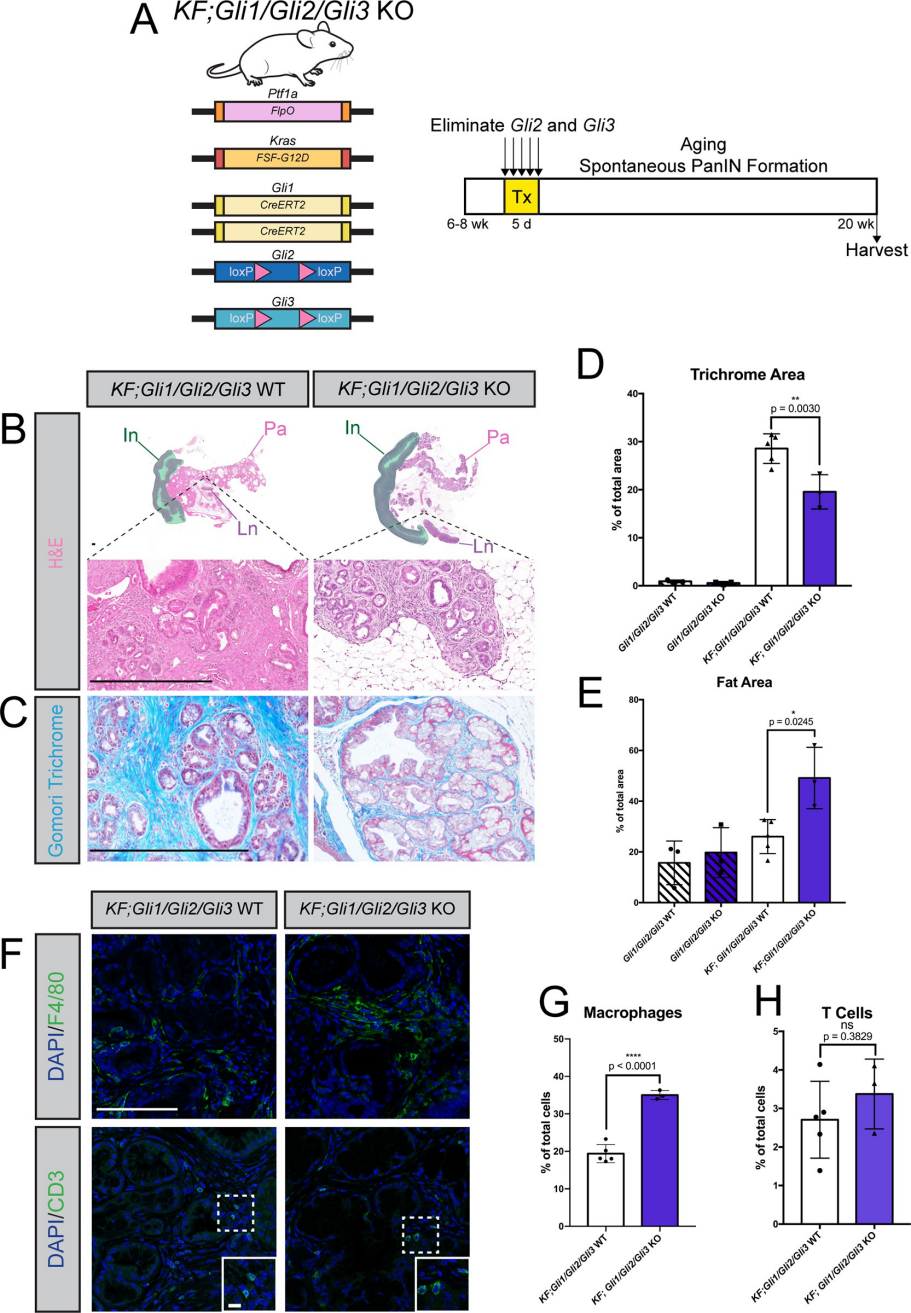
Combined *Gli1-3* deletion drives widespread tissue loss during PanIN progression. **A**) Cartoon depicting experimental strategy. Adult *KF; Gli1*^*CreERT2/CreERT2*^*; Gli2*^*fl/fl*^; *Gli3*^*fl/fl*^ (*KF;Gli1/Gli2/Gli3* KO) mice were given tamoxifen (Tx, 200mg/kg) once a day for 5 days. Pancreata were harvested at 20 weeks of age. **B-E**) Histological analysis of *KF; Gli2*^*fl/fl*^; *Gli3*^*fl/fl*^ (*KF;Gli1/Gli2/Gli3* WT) (left) and *KF;Gli1/Gli2/Gli3* KO (right) mice, including H&E staining (**B**), Gomori trichrome (**C,** quantified in **D**), and fat area (**E**). Green shaded area outlines intestinal tissue (In). Pancreas tissue (Pa) and Lymph nodes (Ln) annotated accordingly. Scale bar = 500μm. P-values determined by ordinary one-way ANOVA with Tukey’s multiple comparisons test. **F-H**) Immunofluorescent antibody detection (**F**) and quantitation (**G-H**) of macrophages (F4/80) and T cells (CD3) in *KF;Gli1/Gli2/Gli3* WT and *KF;Gli1/Gli2/Gli3* KO mice. Inset scale bar = 10μm. All other scale bars = 100μm. P-values were determined by unpaired t-test. N ≥ 3 for all genotypes. Mouse drawing acquired from the open source repository SciDraw.io (doi.org/10.5281/zenodo.3925901).

While validating our *KF;Gli2/Gli3* cKO model, we were surprised to find that *Gli1* expression is maintained following the loss of *Gli2/Gli3* (Fig 3J). This contrasts with *Gli2;Gli3* double mutant embryos, which lack *Gli1* expression across multiple developing tissues [53]. We hypothesized that *Gli1* could be functioning partially redundantly with *Gli2* and *Gli3*, and that deleting all three *Glis* would accentuate the phenotypes we observed in *KF;Gli2/Gli3* cKO mice. To test this, we utilized *Gli1*^*CreERT2*^ mice [54], in which a *CreER-t2* allele knocked into the endogenous *Gli1* locus abolishes *Gli1* expression while driving recombination in pancreatic fibroblasts [50]. Crossing this allele into *KF* mice enabled us to target *Gli2* and *Gli3* expression in *Gli1*-expressing fibroblasts during PanIN progression (Fig 4A). RNAscope analysis of *KF; Gli1*^*CreERT2/CreERT2*^*; Gli2*^*fl/fl*^; *Gli3*^*fl/fl*^ (*KF;Gli1/Gli2/Gli3* KO) mice confirmed complete elimination of *Gli1* (S3A Fig) and efficient conditional reduction of *Gli3* (S3B Fig). Once validated, *KF;Gli1/Gli2/Gli3* KO mice were aged to 20 weeks to evaluate spontaneous PanIN progression in the absence of *Gli1-3*.

Strikingly, combined *Gli1/Gli2/Gli3* depletion leads to a dramatic loss of pancreas parenchyma, with concurrent pancreatic lipomatosis, or adipocyte accumulation in the pancreas (Figs 4B–4E and S3C). Pancreatic lipomatosis is observed following extensive acinar cell death [55,56]. Although no differences in cell death are detected between *KF;Gli1/Gli2/Gli3* WT and *KF;Gli1/Gli2/Gli3* KO tissue at the time of dissection (S3C Fig), the loss of acinar tissue likely occurred earlier in disease progression, resulting in the disrupted state of the pancreas following *Gli* deletion. Interestingly, the pancreata of *Gli1/Gli2/Gli3* KO mice that do not express oncogenic *Kras* are grossly normal, and do not show any evidence of tissue loss or pancreatic lipomatosis (Fig 4D and 4E). Thus, the combined loss of *Gli1/Gli2/Gli3* only compromises tissue integrity in the context of pancreatic carcinogenesis.

In addition to disrupted tissue architecture, the pancreata of *KF;Gli1/Gli2/Gli3* KO mice feature an increase in macrophages compared to *KF;Gli1/Gli2/Gli3* WT mice (Fig 4F and 4G), in contrast to the decrease in macrophages observed in *KF;Gli2/Gli3* cKO tissue (cf. Fig 2E). We did not find any difference in T cell number between *KF;Gli1/Gli2/Gli3* KO and *KF;Gli1/Gli2/Gli3* WT mice (Fig 4F and 4H). Thus, a baseline level of *Gli* activity is necessary to maintain pancreas integrity during PanIN progression. Further, total ablation of *Gli* promotes macrophage infiltration, suggesting that macrophages are sensitive to subtle changes in GLI activity in fibroblasts.

### Loss of *Gli2* and *Gli3* reduces tumor growth through the recruitment of NK cells

**Fig 5:**
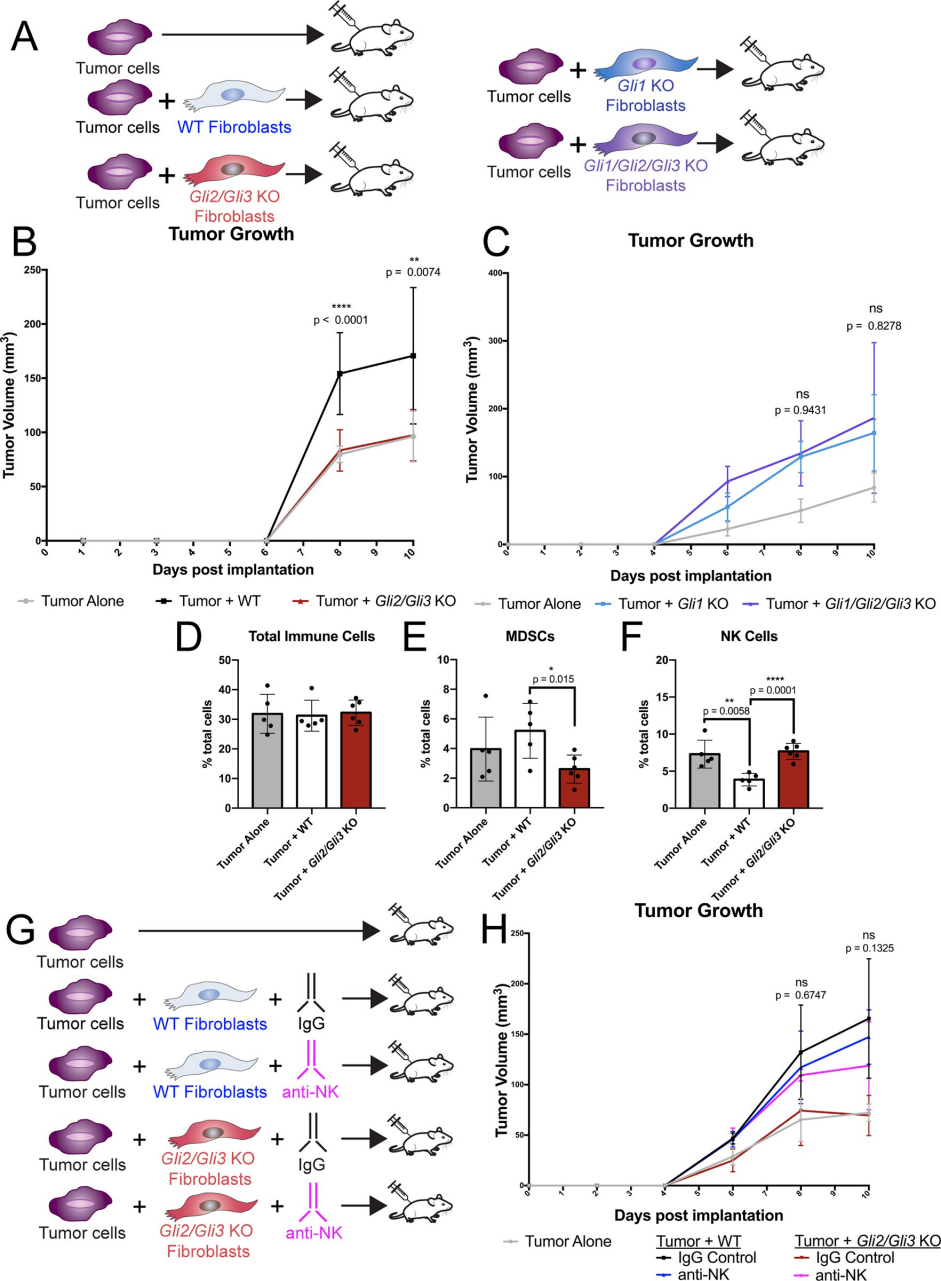
Loss of *Gli2* and *Gli3* reduces tumor growth through the recruitment of NK cells. **A**) Cartoon depicting experimental strategy. Tumor cells were injected subcutaneously either alone or in combination with pancreatic fibroblasts into nude mice. (**B-C**) Volume (mm^3^) of implanted tumors over time. Tumor cells were injected alone (gray), or co-injected with WT (black), *Gli2/3* KO (red), *Gli1* KO (blue), or *Gli1/Gli2/Gli3* KO (purple) pancreatic fibroblasts. Displayed p-values compare *Gli* KO fibroblasts to their corresponding parental line control. (**D-F**) Flow cytometry analysis of total immune cells (**D**), MDSCs (**E**), or NK cells (**F**) in subcutaneous tumors. (**G**) Cartoon depicting experimental strategy for NK cell depletion experiment. Animals were either treated with an IgG control or an anti-NK cell depleting antibody (anti-asialo GM1). (**H**) Volume (mm^3^) of implanted tumors following NK Cell depletion. P-values determined by ordinary one-way ANOVA with Tukey’s multiple comparison test. The displayed p-values compare Tumor + *Gli2/Gli3* KO + anti-NK conditions to Tumor + WT + IgG conditions. For all analyses, n ≥ 5 tumors for each experimental condition. Mouse drawing acquired from the open source repository SciDraw.io (doi.org/10.5281/zenodo.3925901).

To study the role of *Gli1/Gli2/Gli3* in tumor growth, we performed tumor implantation experiments with *Gli* KO pancreatic fibroblasts (Fig 5A). Since germline *Gli2* and *Gli3* mutants die perinatally [57,58], we utilized *Gli*^*fl/fll*^ mice to derive fibroblast lines from the adult pancreas. Once established, fibroblast lines were infected with either a GFP-expressing adenovirus (WT lines) or a Cre-expressing adenovirus (*Gli* KO), and *Gli* deletion was validated by qPCR and western blot (S4A–S4D Fig). Both *Gli2/Gli3* KO and *Gli1/Gli2/Gli3* KO pancreatic fibroblasts are unresponsive to HH stimulation, while parental line controls remain HH responsive (S4E and S4F Fig). *Gli* KO fibroblasts were co-injected with KPC-derived tumor cells [59] into the flanks of nude mice, which lack functional T and B cells. Consistent with previous findings [18,60], co-injecting tumor cells with WT fibroblasts produces larger tumors than tumor cells alone (Fig 5B). In contrast, co-injecting *Gli2/Gli3* KO fibroblasts with tumor cells fails to promote tumor growth, and produces tumors that are significantly smaller than tumors co-injected with WT fibroblasts (Fig 5B). Given the detrimental effects observed when *Gli1*, *Gli2*, and *Gli3* are deleted at PanIN stages (Fig 4), we next tested how *Gli1/Gli2/Gli3* KO fibroblasts impact invasive tumor growth. Strikingly, co-injection of tumor cells with *Gli1/Gli2/Gli3* KO fibroblasts promotes tumor growth to the same degree as parental *Gli1* KO fibroblasts and WT control fibroblasts (Fig 5C, cf. Fig 5B). Thus, while reduction of *Gli* restrains tumor growth, total ablation of *Gli* promotes tumor growth.

We next wanted to determine why *Gli2/Gli3* KO fibroblasts fail to promote tumor growth. To confirm that *Gli2/Gli3* KO fibroblasts persist in this transplantation model, we analyzed harvested tumors for the presence of a tdTomato reporter allele expressed by *Gli2/Gli3* KO fibroblasts. TdTomato expression was detected by IF in tumors from our *Gli2/Gli3* KO fibroblast condition (S5A Fig), confirming that the decrease in tumor growth was not simply due to the death of injected fibroblasts. We next assessed whether *Gli2/Gli3* KO fibroblasts impacted the growth of tumor cells directly. However, we found no differences in tumor cell proliferation nor cell death between *Gli2/Gli3* KO and WT conditions (S5B and S5C Fig). We also did not find any differences between fibroblast nor endothelial cell abundance across our experimental conditions (S5D and S5E Fig). Given the connection between *Gli* expression and immune infiltration that we observed at PanIN stages, we next investigated whether this reduction in tumor growth was due to altered recruitment of immune cells. We found no difference in total immune cells between our different experimental conditions (Fig 5D). However, when we analyzed different subpopulations of immune cells (Figs 5E and 5F, and S5F and S5G), we determined that *Gli2/Gli3* deletion in fibroblasts leads to a decrease in MDSCs and an increase in NK cells (Fig 5E and 5F). Interestingly, *Gli2* KO, *Gli3* KO, and *Gli1/Gli2/Gli3* KO fibroblasts do not impact MDSC nor NK cell infiltration (S5H–S5J and S6A–S6L Figs), indicating that this effect on immune infiltration is specific to *Gli2/Gli3* KO fibroblasts.

We were surprised to find that total myeloid cells and macrophages do not change following the loss of *Gli2/Gli3* in our tumor implantation experiments (S5F and S5G Fig), in contrast to our *KF;Gli2/Gli3* cKO model (cf. Figs 2E and S2E). We wondered if this difference could be due to the different genetic strains between our experimental systems, as *KF;Gli2/Gli3* cKO mice are fully immune competent and possess functional T cells, while nude mice do not. We hypothesized that the loss of *Gli2/Gli3* leads to a decrease in immunosuppressive myeloid cells (MDSCs/macrophages) and an increase in cytotoxic immune cells (T cells/NK cells), and the exact cell types involved depend on the model system. We therefore suspected that in the absence of T cells, the enhanced NK cell recruitment in our *Gli2/Gli3* KO condition was responsible for antagonizing tumor growth. To test whether this infiltration of NK cells suppresses tumor growth, tumor-bearing mice were treated with an NK cell-depleting antibody (anti-asialo GM1; Figs 5G and S6M–S6P). Tumor growth following co-injection with WT fibroblasts is unaffected by NK cell depletion (Fig 5H), presumably due to NK cells already being excluded from the microenvironment. In contrast, depleting NK cells in tumors co-injected with *Gli2/Gli3* KO fibroblasts rescues tumor growth, and the resulting tumors are equivalent in size to tumors co-injected with WT fibroblasts (Fig 5H). These data reveal that the loss of *Gli2/Gli3* in fibroblasts restricts tumor growth through the recruitment of NK cells.

### *Gli* activity in fibroblasts directly controls macrophage and T cell migration

**Fig 6:**
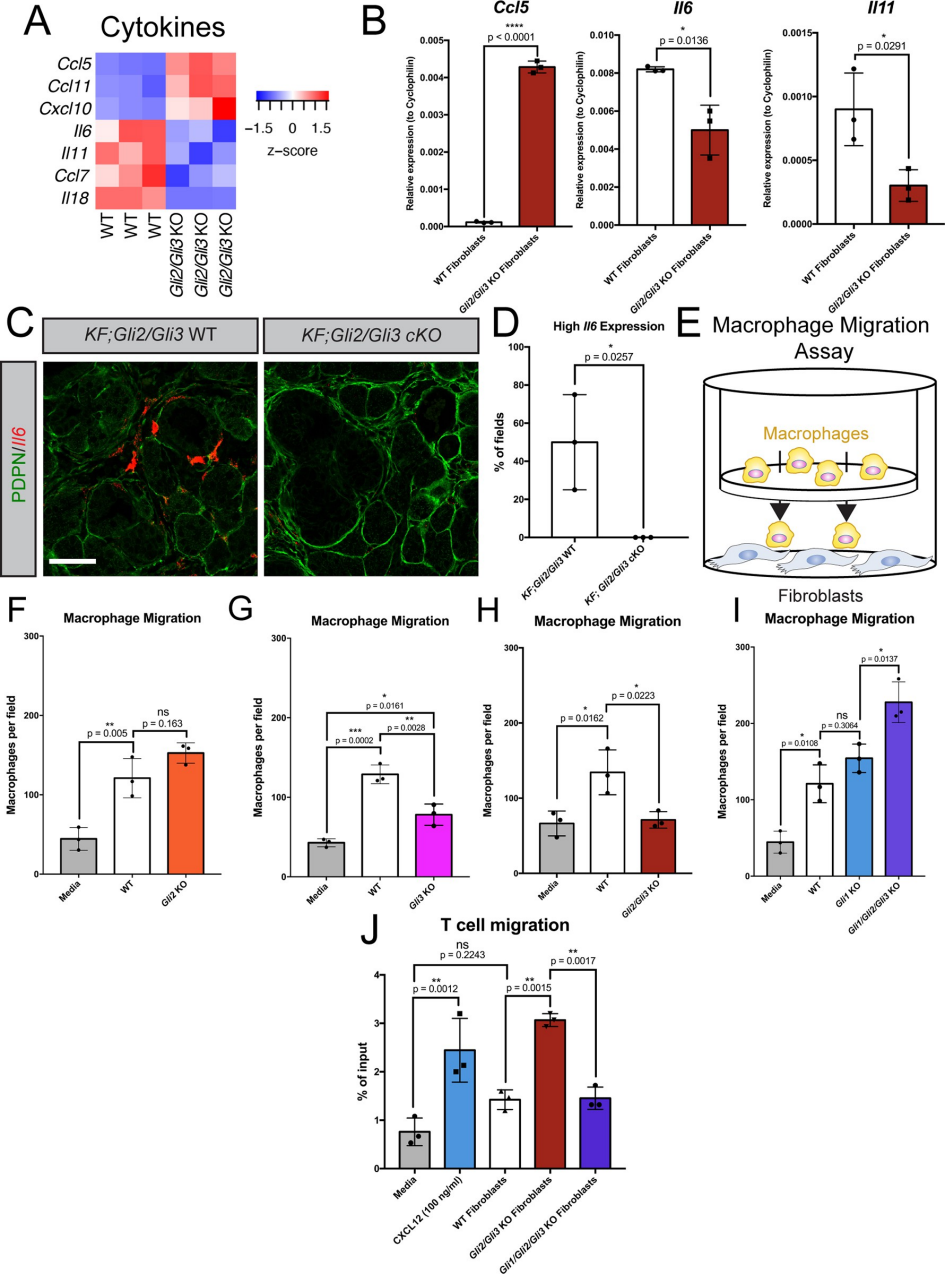
*Gli1*-3 in fibroblasts directly control macrophage and T cell migration. **A**) Differential expression of cytokines in *Gli2/Gli3* WT and *Gli2/Gli3* KO fibroblasts, as determined by RNA sequencing. **B**) qPCR analysis of *Ccl5* (left) and *Il6* (center) and *Il11* (right) in *Gli2/Gli3* WT and *Gli2/Gli3* KO fibroblasts. **C**) *Il6* expression (red) by fibroblasts (PDPN, green) in *KF;Gli2/Gli3* WT and *KF;Gli2/Gli3* cKO mice, as determined by RNAscope. Scale bar = 50 μm **D**) Quantification of high *Il6-*expressing fields of view in in *KF;Gli2/Gli3* WT and *KF;Gli2/Gli3* cKO mice (n = 3 for each genotype). High *Il6* expression defined as an integrated density value (for the *Il6* probe channel) >2300000. P-values for (**B**) and (**D**) determined by un-paired T test. **E**) Cartoon depicting macrophage migration assay experimental strategy. Macrophages and *Gli* KO fibroblasts are separated by an 8μm pore transwell membrane, and macrophages are allowed to migrate through the membrane for 12h. (**F-I**) Quantitation of macrophage migration following co-culture with WT fibroblasts (**F-I**), *Gli2* KO fibroblasts (**F**), *Gli3* KO fibroblasts (**G**), *Gli2/Gli3* KO fibroblasts (**H**), *Gli1 KO* fibroblasts (**I**), and *Gli1/Gli2/Gli3* KO fibroblasts (**I**). **J**) Quantitation of T cell migration following co-culture with WT, *Gli2/Gli3* KO, and *Gli1/Gli2/Gli3* KO fibroblasts. Recombinant CXCL12 (100 ng/ml) added to media was used as a positive control. P-values for (**F-J**) were determined by ordinary one-way ANOVA with Tukey’s multiple comparison test.

To further investigate GLI2/GLI3 function in the pancreatic TME, we transcriptionally profiled *Gli2/Gli3* KO pancreatic fibroblasts by RNA sequencing (RNAseq). RNAseq analysis identified over 2,200 differentially expressed genes in *Gli2/Gli3* KO fibroblasts compared to WT fibroblasts (S1 Supplemental File). When we filtered the data for membrane-bound and secreted factors [61], we detected a number of differentially expressed ECM components and receptors (S7A and S7B Fig), consistent with the impaired ECM deposition we observe following the loss of *Gli2* and *Gli3 in vivo* (cf. Fig 2C). Further, we detected several cytokines that are upregulated in *Gli2/Gli3* KO fibroblasts (Figs 6A and S7A). Specifically, *Ccl5* and *Cxcl10*, two genes encoding T cell and NK cell chemoattractants [62–64], are increased in *Gli2/Gli3* KO fibroblasts (Fig 6A). Further, two myeloid-modulating cytokines, *Il6* and *Il11*, [65,66] are reduced in *Gli2/Gli3* KO fibroblasts (Fig 6A). We validated these changes in cytokine expression by qPCR (Fig 6B). To determine if these transcriptional changes in *Gli2/Gli3* KO fibroblasts are maintained *in vivo*, we analyzed cytokine expression in *KF;Gli2/Gli3* cKO mice by RNAscope. While *Il6* expression in fibroblasts is heterogeneous (as expected from previous work [67]), we detected PDPN+ fibroblasts with high *Il6* expression in our *KF;Gli2/Gli3* WT mice (Fig 6C and 6D). In contrast, no PDPN+ fibroblasts with high *Il6* expression were detected in *KF;Gli2/Gli3* cKO mice (Fig 6C and 6D), indicating that *Gli2* and *Gli3* deletion restricts *Il6* expression *in vivo*.

Although these changes cytokine expression are consistent with the decrease in macrophages and increase in T cells observed *in vivo*, it remained unclear whether *Gli2/Gli3* deletion in fibroblasts impacted the function of these immune cells directly. To investigate the interaction between fibroblasts and macrophages mechanistically, we performed macrophage migration assays [68], in which macrophages were placed above fibroblasts on a transwell membrane, and allowed to migrate for 12 hours (Fig 6E). WT fibroblasts consistently promote macrophage migration compared to media alone (Fig 6F–6I). Loss of *Gli2* alone does not affect macrophage migration (Fig 6F), while loss of *Gli3* alone leads to a partial reduction of macrophage migration (Fig 6G). In contrast, *Gli2/Gli3* KO fibroblasts reduce macrophage migration to near baseline levels (Fig 6H). This reduction in macrophage migration is consistent across multiple macrophage phenotypes, including M0, M1, M2, and TAMs (S7C Fig). Interestingly, *Gli1/Gli2/Gli3* KO fibroblasts promote macrophage migration to a significantly greater degree than either WT or *Gli1* KO parental line control fibroblasts (Fig 6I). These data are consistent with what we observe *in vivo* (cf. Fig 4G), indicating that the increase in macrophage infiltration in *KF;Gli1/Gli2/Gli3* KO mice is directly due to the loss of *Gli1/Gli2/Gli3* in fibroblasts. Further, these *Gli*-dependent effects on macrophages are mediated by secreted factors, as conditioned media from WT and *Gli* KO fibroblasts recapitulate the effects observed in fibroblast co-culture experiments (S7D Fig). Together, our data indicate that *Gli*-mediated cytokine expression directly regulates macrophage infiltration in PDA.

To determine if this effect is conserved in human fibroblasts, we performed macrophage migration assays with human pancreatic stellate cells (hPSCs) [7]. We confirmed that hPSCs are HH-responsive, and upregulate the HH target genes *GLI1* and *PTCH1* in response to HH stimulation (S7E and S7F Fig). In addition, hPSCs promote macrophage migration to a similar degree as WT mouse fibroblasts (S7G Fig). Thus, human pancreatic fibroblasts are both HH-responsive and directly promote macrophage migration.

We next investigated the consequence of fibroblast-specific *Gli* deletion on T cells. We first evaluated whether *Gli* expression regulates T cell differentiation and polarization. Our *in vivo* data indicate that fibroblast-specific *Gli2/Gli3* deletion leads to a subtle (and not statistically significant) increase in regulatory T cells (Tregs, S2H Fig). Since Tregs regulate immune suppression in PDA through cross-talk with fibroblasts [61], we investigated whether loss of *Gli* in fibroblasts impacts Treg differentiation. While T cells co-cultured with *Gli2/Gli3* KO fibroblasts do not significantly increase expression of the Treg marker *Foxp3*, T cells co-cultured with *Gli1/Gli2/Gli3* KO fibroblasts do significantly upregulate *Foxp3* (S8A Fig). These data are consistent with the notion that *Gli1/Gli2/Gli3* KO fibroblasts promote immune suppression, as suggested by enhanced macrophage infiltration both *in vitro* and *in vivo* (Figs 4G and 6I). Notably, the expression of functional Treg markers associated with an immunosuppressive phenotype (including *Il10* and *Tgfb*) is not significantly altered across our different *Gli* KO fibroblast lines (S8B and S8C Fig). These data indicate that fibroblasts regulate T cell differentiation into Tregs in a GLI-dependent fashion, but do not affect the gene expression pattern of established Tregs.

To determine if the loss of *Gli* in fibroblasts directly regulates T cell infiltration, we performed transwell T cell migration assays with our *Gli* KO fibroblast lines. While WT fibroblasts do not impact T cell migration, *Gli2/Gli3* KO fibroblasts promote T cell migration to the same degree as a potent T cell chemoattractant, CXCL12 (SDF1α) (Fig 6J). In contrast, *Gli1/Gli2/Gli3* KO fibroblasts do not promote T cell migration, as the degree of migration is comparable to media alone (Fig 6J). Together, these data corroborate the phenotypes we observe *in vivo*, and reveal that fibroblasts directly regulate the migration of both macrophages and T cells through GLI-dependent expression of cytokines.

## Discussion

**Fig 7:**
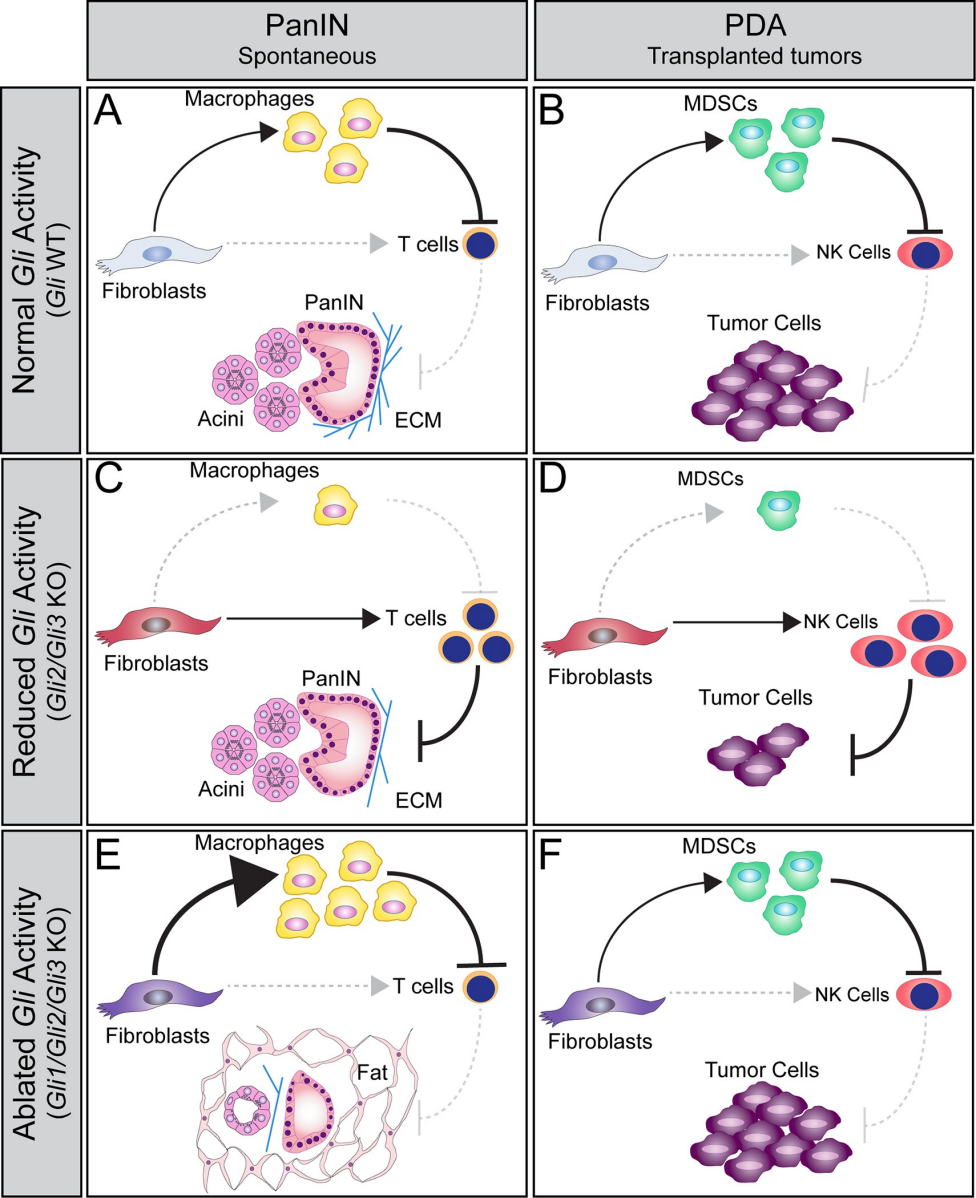
Model of GLI function during PDA progression. (**A-B**) *Gli*-expressing fibroblasts directly promote the recruitment of macrophages and MDSCs (**A,B**, top) at PanIN and PDA stages, respectively. These myeloid cells suppress T cells and NK cells (**A,B**, right), facilitating disease progression. (**C-D**) *Gli2* and *Gli3* deletion in fibroblasts directly reduces the recruitment of myeloid cells, and directly promotes T cell and NK cell infiltration (**C,D**, right). Loss of *Gli2* and *Gli3* also decreases collagen deposition and slows tumor growth (**C,D**, bottom). However, when all three *Glis* are deleted (**E**, **F**), fibroblasts have an enhanced ability to recruit macrophages (**E**, top) and a sustained ability to recruit MDSCs (**F**, top), leading to T cell and NK cell exclusion (**E,F**, right). Thus, *Gli1/Gli2/Gli3* KO fibroblasts support tumor growth (**F**, bottom). Interestingly, loss of *Gli1/Gli2/Gli3* also leads to the loss of pancreas tissue and an accumulation of fat at PanIN stages (**E**, bottom), indicating that a baseline level of GLI activity is necessary to maintain pancreas integrity.

In this study, we investigated the individual and combined roles of *Gli1-3* throughout PDA progression. We determined that *Gli1-3* are expressed by fibroblasts in the healthy pancreas, and that expression of all *Glis* expands in PanIN and PDA stages of disease. Through a combination of genetic mouse models and *ex vivo* approaches, we found that GLIs direct the fibroinflammatory response during PanIN progression and in PDA (Fig 7). Reducing *Gli* activity through loss of *Gli2* and *Gli3* decreases collagen and reduces the infiltration of immunosuppressive myeloid cells, and at the same time promotes T cell infiltration. In a PDA transplantation model, where T cells are absent in the host, we observe an increase in NK cell infiltration, that in turn reduces tumor growth. However, a baseline level of *Gli* activity is necessary, as deleting all three *Glis* leads to a dramatic loss of pancreas tissue, an increase in macrophage infiltration, sustained T cell exclusion, and enhanced tumor growth. Together, these data demonstrate that differing levels of *Gli* activity have opposing functions throughout PDA progression, and that *Gli*-driven changes in immune infiltration determine tumor growth.

### Tumor-supporting and tumor-restricting roles for HH in PDA

Unraveling the role of HH signaling in PDA has proven challenging, as reports have described contradictory roles for the pathway in pancreatic cancer [15,18–20]. However, these seemingly conflicting findings may be an accurate reflection of the complicated and nuanced biology at play in this disease. Our data support a model in which reduced HH signaling promotes tumor growth, while total ablation of HH pathway activity reduces it. Subtle differences in how the pathway is manipulated alters the levels of HH signaling, and tip the scales towards tumor-promoting or tumor-restricting effects.

Our data provide evidence that the activity of multiple transcription factors regulate PDA progression, a theme that has been seen at all levels of the HH signaling pathway. For example, multiple HH ligands (*Shh* and *Ihh*) are expressed in PDA [19,28,35], and while loss of a single ligand promotes tumor growth [19,20], the absence of both *Ihh* and *Shh* reduces tumor growth [35]. Importantly, this decrease in tumor growth is only seen in a HH-sensitized model (when host mice lack one copy of *Gli1*), further demonstrating that severe inhibition of HH, not slight reduction, is necessary to reduce tumor growth. Similarly, multiple HH co-receptors (*Gas1*, *Boc*, *Cdon*) regulate pancreatic tumor growth [18]. While loss of two receptors (*Gas1* and *Boc*) promote tumor growth, deleting all three co-receptors reduces tumor growth [18]. These patterns of tumor growth in *Gas*^*-/-*^*;Boc*^*-/-*^ and *Gas1*^*-/-*^*;Boc*^*-/-*^*;Cdon*^*-/-*^ tumors also coincide with increased and decreased vasculature, respectively, indicating that subtle differences in HH signaling levels impact multiple compartments within the TME. Together, these data from throughout the pathway indicate that slight reduction of HH signaling promotes disease progression, while severe inhibition restrains it.

Taken alone, the reduction of tumor growth following severe inhibition of HH signaling would indicate that HH solely supports tumor growth. However, our data also indicate that activation of HH can be protective. Loss of GLI repressor (via *Gli3* deletion) reduces the migration of macrophages and decreases tumor growth, demonstrating that HH activation can antagonize PDA. This finding is consistent with previous work, in which pharmacological activation of HH signaling via Smoothened agonist (SAG21k) led to decreased proliferation/abundance of PanIN lesions [20]. Thus, HH has the ability to both promote and restrict tumor growth, and the net effect depends on the levels of HH pathway activity.

Beyond the importance of signaling levels, HH pathway components have functions outside of canonical signal transduction. While the primary role of GLIs is to regulate levels of HH signaling, growing evidence indicates that GLIs also influence PDA through HH-independent mechanisms. Although the canonical HH response is restricted to fibroblasts, tumor cells can aberrantly activate GLIs. Non-canonical upregulation of GLI2 causes tumor cells to adopt a more basal subtype, leading to an increase in mesenchymal markers and a decrease in epithelial markers [69]. This finding is consistent with previous work from our group, where ectopic expression of constitutively active GLI2 drove the formation of undifferentiated tumors [70]. Conversely, antagonizing GLI targets in tumor cells either by knocking down *Gli1* [41] or over-expressing a constitutive GLI3 repressor [71] increases tumor cell death and reduces colony formation. Thus, GLI activity can promote tumor growth in epithelial cells in a cell-autonomous, HH-independent manner.

While aberrant upregulation of GLI promotes tumor cell growth, our single-cell and laser capture RNA sequencing analysis indicate that GLIs are predominantly expressed in the stroma. Therefore, in this study we focused our attention on the role of GLI1-3 in the stroma. However, even within the stroma there are non-canonical functions for HH pathway components. For example, genetic deletion of *Smo* in pancreatic fibroblasts eliminates the HH-response, but leads to the aberrant activation of AKT [72,73]. AKT is then able to stabilize GLI2 in fibroblasts, which in turn promotes epithelial cell growth via TGF-α secretion [72]. These HH-independent roles for GLIs open up the possibility that the phenotypes we observe in *Gli* KO fibroblasts could be due to a combination of both canonical as well as non-canonical GLI functions. Exploring this possibility requires a deeper investigation into GLI targets in PDA.

Here we describe the coordinated roles of all three GLIs *in vivo* within the context of PDA progression. Our data demonstrate that manipulating GLI has both tumor-promoting and tumor-restricting effects, depending on their combinatorial activity. However, the transcriptional mechanisms driving these different phenotypes remain unclear. Our RNA sequencing analysis of *Gli2/Gli3* KO fibroblasts indicates that the coordinated activity of GLI2 and GLI3 drive a transcriptional program that shapes the extracellular and immune landscape of PDA. Further, pervious work has identified a number of transcriptional targets of GLI1 in pancreatic fibroblasts, including *Il6*, *Il8*, *Mcp-1*, *M-csf* [39,48]. However, the degree of overlap between GLI1, GLI2, and GLI3 transcriptional targets in PDA is still unknown. In addition, it is possible that GLI target genes may change at different stages of disease progression. Fortunately, the development of ChIP-capable tags on endogenous *Gli* alleles (e.g., 3x*Flag-Gli3*) [74] provides an opportunity to define GLI target genes *in vivo*. Future studies could utilize ChIP-capable GLI1-3 proteins to define shared and unique GLI target genes, and evaluate how GLI-driven transcriptional programs change throughout PDA progression. Building out this transcriptional roadmap could help identify the HH targets responsible for driving tumor-promoting versus tumor-restricting programs in PDA, opening up new, more targeted avenues for potential therapies.

### Hedgehog-Immune crosstalk

Fibroblasts play a crucial role in regulating immune infiltration in PDA, and are essential in driving immune-suppression [13,14,61]. Further, growing evidence supports the notion that HH signaling regulates immune infiltration in pancreatic cancer. Prior work has demonstrated that *Gli1* drives the expression of immune-modulatory cytokines [39,48]. More recently, pharmacological inhibition of SMO (via LDE225) in tumor-bearing mice was shown to increase the recruitment of immunosuppressive macrophages and decrease the relative proportion of cytotoxic T cells [35]. Our data confirms that disrupting HH signaling dramatically alters immune infiltration in the context of PDA progression. However, combined loss of *Gli2* and *Gli3* decreases the recruitment immunosuppressive macrophages and increases the recruitment of cytotoxic T cells. At first glance these results seem surprising, as both LDE225-treated and *Gli2/Gli3* KO fibroblasts do not respond to HH. This discrepancy raises the question: why does SMO inhibition and *Gli* deletion drive such divergent immune phenotypes?

One essential difference between these two experimental systems is the combinatorial activity of the GLIs. Although *Gli2/Gli3* KO pancreatic fibroblasts do not upregulate target genes following HH stimulation, we found that a baseline level of *Gli1* expression is maintained in these cells. As a result, some GLI target genes could still be bound by this baseline level of GLI-activator in the absence of *Gli2* and *Gli3*. In contrast, LDE225 treatment effectively eliminates *Gli1* expression in pancreatic fibroblasts [35], leading to a fully “HH Off” state. Thus, genetic loss of *Gli2* and *Gli3* represents a different level of HH pathway activity compared to LDE225-treated pancreatic fibroblasts. Given the evidence that different levels of HH activity drive radically different phenotypes in PDA [18], it is not surprising that we see distinct immune phenotypes between *Gli2/Gli3* cKO and LDE225-treated mice. Notably, when we eliminate all three *Glis*, we observe patterns of immune infiltration that are more consistent with LDE225 treatment, presumably due to the elimination of redundant GLI-activator.

Beyond the compensatory actions of GLI-activators, *Gli2/Gli3* KO fibroblasts fundamentally differ from LDE225-treated cells due to the loss of *Gli3*. We see in our system that loss of *Gli3* alone is sufficient to partially reduce the migration of macrophages. This indicates that in the context of PDA progression, de-repression of GLI target genes is an important force in regulating immune infiltration. In our *Gli2/Gli3* KO fibroblasts, the absence of a repressor combined with the presence of an activator (GLI1) has the potential to drive substantial transcriptional activity, even in the absence of a HH response. Together, these data emphasize the importance of combinatorial GLI activity in regulating disease progression, and reveal how subtle differences in HH transcriptional activity can dramatically shape the immune landscape of PDA. These findings also provide further rationale for defining GLI-activator versus GLI-repressor transcriptional targets throughout PDA progression.

In addition to demonstrating the differences between pharmacological and genetic manipulation of HH signaling, these studies reveal how the role of HH in regulating immune infiltration changes at different stages of disease. In the present study, *Gli* was deleted in fibroblasts prior to the formation of PanIN lesions (KF mice) or before exposure to tumor cells (tumor implantation experiments). Therefore, our data reveal how the immune landscape of PDA develops in the absence of normal HH activity. In contrast, pharmacological inhibition in tumor-bearing mice demonstrate the impact of removing HH signaling from an established disease [35]. Prior research has demonstrated that disrupting HH signaling in fibroblasts has different consequences on PDA progression depending on the stage of disease studied [48,75]. It is therefore likely that the role of HH on the immune system also evolves throughout PDA progression, and that some of the differences we observe reflect a shift in HH’s role. Fortunately, the inducible nature of the *KF; Pdgfrα*^*CreER-T2*^*;Gli2*^*fl/fl*^; *Gli3*^*fl/fl*^ mouse model could be leveraged to delete *Gli* after the formation of PanIN lesions, directly testing the role of *Gli* in established pancreatic disease. Thus, this experimental system provides new opportunities to evaluate the role of HH signaling at multiple stages of PDA.

Although our study provides new insight into *Gli*-mediated regulation of immune infiltration in PDA, many open questions remain. One outstanding question is how GLIs regulate the balance of CAF subtypes in PDA. A growing body of work is revealing the heterogeneity of pancreatic fibroblasts, and demonstrating that different populations have distinct roles in the context of pancreatic cancer [23,67,76,77]. Further, pharmacological inhibition of HH changes the balance of inflammatory CAFs (iCAFs) and myofibroblastic CAFs (myCAFs) *in vivo*, resulting in a more immunosuppressive microenvironment [35]. Given the ability of GLIs to fine-tune HH responses in pancreatic fibroblasts, a natural next question is how different GLIs influence the relative proportion of CAFs in PDA. Our expression analysis *in vitro* and *in vivo* demonstrates that loss of *Gli2/Gli3* reduces *Il6* expression, a key marker of iCAFs. A reduction in iCAFs would be consistent with the reduction in immunosuppressive immune cells that we observe *in vivo*. However, more in-depth analysis will be necessary to explore the potential connections between GLIs and fibroblast heterogeneity.

Overall, our data indicate that all three GLIs play a central role in PDA progression. The reduction in immunosuppression following *Gli2/Gli3* deletion warrants further exploration into the transcriptional networks downstream of *Gli*. Identifying and targeting these mechanisms of immunosuppression could provide new avenues for future therapies, potentially enhancing the efficacy of immunotherapy in this challenging disease.

## Materials and methods

### Ethics statement

All experiments performed in this study were approved by the University of Michigan Institutional Animal Care and Use Committee (IACUC), the University of Michigan Institutional Biosafety Committee (IBC), and the University of Michigan Institutional Review Boards (IRB).

### Laser-capture microdissection and RNA sequencing (LCM-RNAseq)

LCM-RNAseq was performed and analyzed by Maurer and colleagues [40]. Briefly, samples underwent laser capture microdissection using a PALM MicroBeam microscope (Zeiss). RNA libraries were generated using the Obation RNAseq System V2 kit (NuGEN), and sequenced to a depth of 30 million, 100 bp, single-end reads.

### Single-cell RNA sequencing (scRNAseq)

scRNAseq data were generated and processed as previously described [42]. Briefly, processed data were normalized using the Seurat (V4) [78] pipeline in R with a scale factor of 10,000 and the LogNormalize normalization method. Variable genes were identified using FindVariableFeatures. Data were scaled and centered using linear regression and principal component analysis (PCA) was run with the RunPCA function using the defined variable genes. Genes in the HH pathway were displayed as a Dot Plot analysis.

### X-gal staining

Pancreata were dissected in chilled 1x PBS (pH 7.4). Tissue samples were collected for RNA isolation and histology, and the remaining tissue was fixed (4% PFA) on ice for 1h. Pancreata were washed 3x5min in PBS and transferred to a PBS+30% sucrose solution overnight at 4°C. The next day, half of the 30% sucrose was removed and replaced with OCT embedding medium, and placed on a rocker at 4°C for 1h. This process was repeated twice, and then the tissue was transferred to 100% OCT for 1h. Tissues were embedded in OCT and sectioned on a Leica CM1950 cryostat (12μm sections). β-Galactosidase activity was detected with X-gal staining solution [5 mM K3Fe(CN)6, 5 mM K4Fe(CN)6, 2 mM MgCl2, 0.01% Na deoxycholate, 0.02% NP-40, 1 mg/ml X-gal] and stained for 2 – 36h at 37°C. After staining, the sections were washed 3x5min in PBS and counterstained with Nuclear Fast Red for 5min. Sections were dehydrated 70% ethanol, 95% ethanol, 100% ethanol and 100% xylene) and mounted with coverslips using Permount Mounting Medium (Thermo Fisher Scientific).

### Immunofluorescence

Tissues were dissected/processed as described above. Frozen sections were warmed to room temperature (RT), then baked at 60°C for 10min. Sections were washed 3x5min in PBS and blocked in blocking buffer [3% bovine serum albumin, 1% heat-inactivated sheep serum, 0.1% Triton X-100 in PBS] for 1h at RT. Paraffin sections were rehydrated (100% xylene, 100% ethanol, 95% ethanol, DI water) and underwent citric acid antigen retrieval (Vector Laboratories, H-3300) for 10min at 92°C. Paraffin sections were washed 3x5min with DI water, 3x5min with PBS, and blocked for 1h at RT in PBS+1% BSA. All sections were incubated with primary antibodies overnight at 4°C in a humidified chamber. Primary antibodies are listed in S1 Table. Secondary antibodies were diluted in blocking buffer and incubated for 1h at RT, followed by 3x5min washes in PBS. All secondary antibodies were used at a 1:500 dilution. Nuclei were labeled with DAPI for 10min at RT. Slides were mounted with coverslips using Immu-mount aqueous mounting medium (for frozen sections) or Permount Mounting Medium (for paraffin sections). Sections were visualized on a Leica SP5X upright confocal or a Nikon E800 epifluorescent microscope. For quantitation, 3–5 fields of view were imaged per section and analyzed using FIJI (version 2.0.0-rc-69/1.52p).

### Subcutaneous tumor growth assays

1 x 10^5^ 7940b tumor cells were mixed with 5 x 10^5^ fibroblasts, resuspended in a 50:50 mix of serum-free media [DMEM+1% Pen/Step] and Matrigel (Corning 354234). Two fibroblast clones (in equal numbers) were used in each injection to reduce the impact of clonal variability. Cells were injected subcutaneously into the flanks of NU/J mice (Jackson Laboratory Stock No: 002019). Tumors were measured every other day with calipers, and animals were sacrificed after 10 days. For NK cell depletion experiments, 10μl of anti-asialo GM1 (Wako 986–10001) or an equivalent volume of normal Rabbit IgG control (R&D AB-105-C) was diluted 1:10 in sterile PBS and injected intraperitoneally. Injections were given 24h before tumor implantation, on the day of tumor implantation, and once every three days for the remainder of the experiment.

### Flow cytometry

Single-cell suspensions of tissue were prepared as previously described [79]. Flow cytometry was performed either on a BioRad Ze5 Analyzer or a MoFLo Astrios cell sorter, and data were analyzed with FlowJo v10 Software. Values for all flow cytometry data displayed as a percentage of total cells. Antibodies used for flow cytometry are listed in S2 Table.

### Histology

Tissue samples were fixed in 10% neutral buffered formalin (Thermo Fisher 245–685) overnight at RT. Samples were washed 3x5min in PBS, moved to 70% ethanol, and then processed for paraffin embedding. 5μm sections were collected and used for histological analysis. Hematoxylin and eosin (H&E) and Gomori Trichrome stain were performed according to standard protocols.

### Macrophage migration assays

Bone marrow cells were isolated as described previously [80] and plated in a 50–50 mixture of complete media [DMEM F12 + 10% Calf Serum + 1% Pen/Step] and tumor cell (7940b) conditioned media. Cells were supplemented with 750μl of 50–50 media every other day for a total of 6 days. On day 5, 2.5 x 10^5^ total fibroblasts were plated into each well of a 12-well plate. Two fibroblast clones (in equal numbers) were used for all conditions. Once cells adhered to the plates (after 8h), the media was replaced with low (0.1%) serum media. On day 6, macrophages were removed from plates with 0.25% Trypsin-EDTA (Gibco 25200–056) and scraping, and resuspended in low serum media. 4 x 10^5^ macrophages were plated onto each transwell insert (8μm pore size, Thermo Scientific 140656) above fibroblast wells. After 12h, remaining macrophages were removed from the top of the transwells with a cotton swab, and the membranes were fixed (4% PFA) for 10min at RT, followed by 3x5 min PBS washes. Membranes were stained with DAPI for 10min at RT, washed 3x5 min in PBS, and removed from the transwells with a scalpel. Membranes were then mounted onto slides with Immu-mount aqueous mounting medium, coverslipped, and imaged as described above.

### T cell differentiation and migration assays

1 x 10^5^ total fibroblasts were plated into each well of a 24-well plate in complete media [DMEM + 10% CS + 1% Pen/Strep]. Two fibroblast clones (in equal numbers) were used for all conditions. 8 h later, the media was replaced with 1% serum media [DMEM + 1% CS + 1% Pen/Strep]. The next morning, single cell suspensions were made from the spleens of BL6 mice, and total T cells were isolated by MACS according to the manufacturer’s instructions (Miltenyi Biotec 130-042-401). T cells in suspension were bound by Biotin-conjugated anti-CD3 antibody (R&D BAM4841), and captured in a magnetic column with anti-Biotin microbeads. Isolated T cells were resuspended in 1% serum media and 2.5 x 10^5^ T cells were added to the top of each transwell. For differentiation assays, 0.4μm pore transwell membranes were used. For migration assays, 5μm pore transwell membranes were used. 100 ng/ml CXCL12 (SDF1α, R&D 460-SD-010) added to the bottom chamber was used as a positive control in migration assays. Plates were returned to the cell culture incubator under standard cell culture conditions for 6.5 h. At the end of differentiation assays, T cells were collected from the top chamber and lysed for RNA isolation (see below). At the end of migration assays, migrated T cells were collected from the bottom chamber, spun down, and counted with a hemocytometer.

### RNAscope

RNAscope was performed as described previously [81]. Briefly, paraffin sections were baked at 60°C for 1h, and then stored overnight at RT. Fluorescent RNAscope was performed according to the manufacturer’s protocol (ACD: 323100-USM). Samples underwent antigen retrieval for 15min, followed by a 12min protease digestion. TSA fluorophores (Akoya biosciences NEL744001KT and NEL745E001KT) were diluted 1:2000 in TSA dilution buffer. Following HRP blocking, slides were washed 3x5 min in PBS and blocked in 0.1% PBS-Tween20 + 5% Normal Donkey Serum for 1h at RT. Primary antibody incubation, secondary antibody incubation, and the subsequent processing, imaging, and quantitation was performed as described above.

### Western blot analysis

20μg of protein was separated on an SDS-polyacrylamide gel (5% GLI2, 7.5% GLI3) for 30min at 80V followed by 90min at 100V. Gels were transferred to an Immuno-Blot PVDF membrane (Bio-Rad; Cat #1620177), blocked with western blocking buffer (30g BSA, 2ml 10% NaN3, Q.S. 1L TBST) for 5min, and probed with antibody diluted in western blocking buffer. Membranes were washed for 3x5min in TBST and then probed with secondary antibody for 1h at RT. Membranes were then washed 12x5min at RT. Protein was detected by fluorescence, using ECL Primer Western Blotting Detection Reagents (RPN2232) developed on a Konica Minolta SRX-101A Medical Film Processor. All primary and secondary antibodies are listed in S3 and S4 Tables, respectively.

### Cell culture

All cell lines were maintained at 37°C and 5% CO_2_, and were kept in standard media (DMEM + 10% Calf Serum + 1% Pen/Strep) unless noted otherwise. The human pancreatic stellate cell (hPSC) line has been previously published [7], and was generously provided by C.A. Lyssiotis. Mouse fibroblast lines were established through the outgrowth method [82]. Briefly, pancreata were isolated from adult mice under sterile conditions. Pancreata were minced mechanically and digested for 15min at 37°C in 1 mg/ml collagenase (Sigma C9263, diluted in sterile Hank’s Balanced Salt Solution—HBSS). Standard media was added to inactivate the enzymatic digestion, and samples were passed through a 70μm cell strainer, resuspended in standard media, and plated onto tissue culture plates coated in 0.1% gelatin. Primary cells (less than 3 passages) were frozen and stored, and kept separate from immortalized lines. Following immortalization, fibroblasts were infected with adenovirus (Control: Ad5 CMV-eGFP and Lenti dsRed; cKO: Ad5 CMV-eGFP and Ad5 CMV-Cre) at an MOI of 500–2000. Successfully infected cells were isolated by flow cytometry and screened by qPCR and western blot for recombination efficacy. Cell lines were tested for mycoplasma before running experiments. For HH signaling assays, 5 x 10^5^ fibroblasts were plated in each well of a 6-well plate in standard media. 24h after plating, the media was replaced with low (0.1%) serum media. 24h later, the media was replaced with 1.5ml of low serum media + 600 nM of Smoothened agonist (SAG) or vehicle control (DMSO). 24h later, a supplemental dose of SAG/vehicle was added directly to each well to a final concentration of 600 nM. 24h later, the cells were lysed and analyzed for HH target gene expression.

### RNA isolation

Snap-frozen tissue samples were pre-treated with RNA*later*-ICE (Invitrogen AM7030) according to the manufacturer’s instructions. Tissue samples and bulk RNA sequencing samples were lysed in Buffer RLT + 1% BME and processed with the Qiagen RNeasy Mini Kit (Qiagen 74104) according to the manufacturer’s instructions. For all other applications, RNA isolation was performed using the PureLink RNA Mini Kit (Invitrogen 12183025) according to the manufacturer’s instructions. All samples were eluted in ultrapure water, and RNA quality was determined using a NanoDrop One (Thermo Scientific ND-ONE-W).

### qPCR

cDNA was generated from 0.5–2μg of RNA using the Applied Biosystems High Capacity cDNA Reverse Transcription Kit (Thermo Fisher 4368814) according to the manufacturer’s instructions. qPCR reactions were run using PowerUp SYBR Green Master Mix (Applied Biosystems A25742) and the primers listed in S5 Table in a StepOnePlus Real-time PCR System (Applied Biosystems 4376600). Gene expression was normalized to Cyclophilin unless stated otherwise. Relative expression was calculated using the 2(^−ddCT^) method.

### Animal models

All mice were housed in specific pathogen-free facilities at the University of Michigan. *Gli1*^*lacZ*^ [46], *Gli2*^*lacZ*^ [37], *Gli3*^*lacZ*^ [47], *Ptf1a*^*Cre*^ (*p48*^*Cre*^) [83], *Kras*^*LSL-G12D*^ [43], *Ptf1a*^*FlpO*^ (*p48*^*FlpO*^) [84], *Kras*^*FSF-G12D*^ [85], *Pdgfrα*^*CreERt2*^ [49], *Gli2*^*fl/fl*^ [86], *Gli3*^*fl/*fl^ [87], *LSL-*tdTomato [88], *Gli1*^*CreERt2*^ [54] mice have all been described previously. To induce Cre recombination, tamoxifen (Sigma T5648) was administered to mice at a dose of 200 mg/kg once per day for 5 days by oral gavage. To induce acute pancreatitis in 6–8 week old KC and KF mice, 8 hourly i.p. injections of caerulein (Sigma C9026) were administered at a dose of 75μg/kg over two consecutive days. Caerulein-treated mice were harvested 3 weeks after their final dose. KF mice used in aging experiments were not given caerulein, and were dissected once the mice reached 20 weeks of age. KPC mice were monitored daily by abdominal palpitation and dissected once the mice reached humane endpoint.

### Statistical analysis

All statistical analysis was performed using Graphpad Prism software. For quantitative analysis, each data point represents an independent biological replicate. Information such as sample size, P value, and the statistical test used is stated in the figure legend. Significant P-values are indicated with one or multiple asterisks according to the following convention: * = p ≤ 0.05, ** = p ≤ 0.01, *** = p ≤ 0.001, **** = p ≤ 0.0001, ns = p > 0.05.

## Supporting information

S1 Fig Characterization of *Gli* expression during human and mouse PDA progression. (**A-C**) Epithelial vs. Stromal expression of HH ligands (**A**) and receptors (**B-C**) in human IPMN (Green, n = 19 Epithelial samples, n = 12 Stromal Samples), PanIN (Blue, n = 26 Epithelial samples, n = 23 Stromal Samples), and PDA (Red, n = 197 Epithelial samples, n = 124 Stromal Samples) tissue, as determined by laser capture microdissection-RNA sequencing (40). (**D-J**) Immunofluorescent antibody analysis of healthy (**D-E**), PanIN (**F-I**), and tumor-bearing (**J**) *Gli-lacZ* reporter mice (n ≥ 3 for all genotypes). Antibodies detect β-Galactosidase (β-GAL, green), fibroblasts (VIM or PDGFβ, Red,**F, J**), ductal cells/PanIN (CK19, Red, **D, G**), blood vessels (CD31, Red, **E, H**), and immune cells (CD45, Red, **I**). DAPI staining in blue. Scale bar = 20μm.
(TIF)

S2 Fig Validation and immune characterization of *KF;Gli2/Gli3* cKO mouse model. **A**) Immunofluorescent antibody detection of a tdTomato reporter (Red) and ECAD (Green) in *KF;Pdgrα*^*CreER/+*^*;LSL-tdTomato/+* mice treated with either tamoxifen (Bottom, n = 4) or vehicle (Top, n = 2). **B**) Efficiency of *Gli2* (Left) and *Gli3* (Right) deletion in *KF;Gli2/Gli3* cKO mice, as determined by RNAscope. Puncta of *Gli* expression were counted and normalized to stromal area in each image. N ≥ 5 for each genotype. **C**) Proliferation (PHH3+) analysis of fibroblasts (PDGFRβ+) in *KF;Gli2/Gli3* cKO and *KF;Gli2/Gli3* WT mice (n ≥ 4 for each genotype). For (**B**) and (**C**), each point represents the average value for an animal, calculated from four independent fields of view. **D**) Immunofluorescent antibody detection of total immune cells (CD45), macrophages (F4/80), total T cells (CD3), and CD8+ T cells (CD8) in *KF;Gli2/Gli3* WT (Top) and *KF;Gli2/Gli3* cKO (Bottom) mice. DAPI staining in blue. Scale bar for all images = 50μm. E-J) Flow cytometry analysis of myeloid cells (**E**), MDSCs (**F**), NK cells (**G**), regulatory T cells (**H**), T helper 2 cells (IL4+) (**I**), and T helper 1 cells (IFNγ+) (**J**) in *KF;Gli2/Gli3* WT and *KF;Gli2/Gli3* cKO mice. For all immune analyses, n ≥ 3 for each genotype. For all quantitation, p-values were determined by un-paired t-test.
(TIF)

S3 Fig Validation and tissue analysis of *KF;Gli1/Gli2/Gli3* KO mice. **A-B**) RNAscope analysis of *Gli1* (**A**, Yellow) and *Gli3* (**B**, Yellow) expression in *KF;Gli1/Gli2/Gli3* WT (Top) and *KF;Gli1/Gli2/Gli3* KO (Bottom) mice. E-Cadherin (ECAD) expressing epithelial cells in red. **C**) Immunofluorescent antibody detection of acinar cells (AMY, Red), PanIN lesions (CK19, Green), and cell death (CC3, Green) in *KF;Gli1/Gli2/Gli3* WT (Top) and *KF;Gli1/Gli2/Gli3* KO (Bottom) mice. DAPI staining in blue. Scale bar = 100 μm. N ≥ 3 for all genotypes.
(TIF)

S4 Fig Validation and HH-responsiveness of *Gli* KO pancreatic fibroblast. **lines A-B**) qPCR analysis of *Gli2* and *Gli3* expression in WT (**A**, blue bars) *Gli2/Gli3* KO (**A**, red bars), *Gli1* KO (**B**, white bars) and *Gli1/Gli2/Gli3* KO (**B**, red bars) pancreatic fibroblast lines. **C-D**) Western Blot analysis for GLI2 (**C**) and GLI3 (**D**) in *Gli* WT and *Gli* KO pancreatic fibroblast lines. Vinculin was used as a loading control. **E-F**) qPCR analysis for HH target genes *Gli1* and *Ptch1* in *Gli2/Gli3* KO (**E**) and *Gli1/Gli2/Gli3* KO (**F**) pancreatic fibroblasts following treatment with vehicle or SAG (600nM). P-values determined by ordinary one-way ANOVA with Tukey’s multiple comparison test.
(TIF)

S5 Fig Additional analysis of *Gli2/Gli3* KO and *Gli1/Gli2/Gli3* KO subcutaneous tumor growth experiments. (**A**) Immunofluorescent antibody detection of a reporter allele (tdTomato, Red) expressed by *Gli2/Gli3* KO pancreatic fibroblasts. Additional antibodies detect fibroblasts (PDGFRα, Green). DAPI staining in blue. Scale bar = 50 μm. (**B-E**) Quantitation of tumor cell proliferation (**B**), cell death (**C**), fibroblast number (**D**), and endothelial cell number (**E**) across experimental conditions. (**F-G**) Flow cytometry analysis of myeloid cells (**F**) and macrophages (**G**) from *Gli2/Gli3* KO subcutaneous tumors. (**H-J**) Flow cytometry analysis of total immune cells (**H**), MDSCs (**I**), and NK cells (**J**) from *Gli1/Gli2/Gli3* KO subcutaneous tumors. For all analyses, n ≥ 5 tumors for each experimental condition. Significance was determined by ordinary one-way ANOVA with Tukey’s multiple comparisons test.
(TIF)

S6 Fig Analysis of tumor growth and immune infiltration in *Gli2* KO, *Gli3* KO, and NK cell depletion subcutaneous tumor growth experiments. (**A-L**) Analysis of tumor implantation experiments incorporating *Gli2* KO (**A-F**), *Gli3* KO (**G-L**) pancreatic fibroblasts. (**A, G**) Tumor volume (mm^3^) over time for *Gli2* KO (**A**) and *Gli3* KO (**G**) tumors. The displayed p-value compares *Gli* KO fibroblasts to their corresponding parental line control. (**B-F, H-L**) Flow cytometry analysis of total immune cells (**B, H**), myeloid cells (**C, I**), macrophages (**D, J**), MDSCs (**E, K**), and NK cells (**F, L**) from subcutaneous tumors. (**M-P**) Further analysis of NK-cell depletion experiments. **M**) Validation of NK cell depletion in anti-NK (anti-asialo GM1)-treated mice compared to IgG control mice. P-value determined by unpaired t-test. **N-P**) Flow cytometry analysis of total immune cells (**N**), macrophages (**O**), and MDSCs (**P**). For all flow cytometry data, values displayed as a percentage of total cells. For all analyses, n ≥ 3 samples for each experimental condition. For all analyses (except **M**), p-values were determined by ordinary one-way ANOVA with Tukey’s multiple comparison test.
(TIF)

S7 Fig Loss of *Gli* alters the transcriptional profile of pancreatic fibroblasts and impacts fibroblast-immune cross-talk. **A-B**) RNA sequencing analysis of *Gli2/Gli3* KO pancreatic fibroblasts and *Gli2/Gli3* WT pancreatic fibroblasts. **A**) Top upregulated (left) and downregulated (right) genes of membrane-bound and secreted proteins. Green gene names indicate ECM genes, and orange gene names indicate cytokines. **B**) Curated gene expression lists of ECM components (left) and ECM receptors (right). **C**) Migration of different macrophage phenotypes (TAM, M0, M1, M2) when co-cultured with WT or *Gli2/Gli3* KO pancreatic fibroblasts. **D**) Macrophage migration following co-culture with pancreatic fibroblast-conditioned media. **E-F**) RT-qPCR analysis of HH target genes (*GLI1*, *PTCH1)* in human pancreatic stellate cells (hPSCs) following stimulation with SAG. Gene expression levels are relative to *GAPDH*. **G**) Macrophage migration following co-culture with media alone, WT mouse fibroblasts, or hPSCs. P-values for (**E-F**) calculated by un-paired t test. All other P-values were calculated by ordinary one-way ANOVA with Tukey’s multiple comparison test.
(TIF)

S8 Fig *Gli* expression in pancreatic fibroblasts regulates Treg differentiation. **A-C**) RT-qPCR analysis of T cells following transwell co-culture with pancreatic fibroblasts. Target genes include markers for Treg identity (*Foxp3*, **A**) as well as markers associated with an immunosuppressive Treg phenotype (*Il10* and *Tgfb*, **B** and **C**, respectively). Gene expression levels are relative to *Cyclophilin*. P-values were determined by ordinary one-way ANOVA with Dunnett’s multiple comparison test.
(TIF)

S1 Table Antibodies used for immunofluorescence. (PDF)

S2 Table Antibodies used for flow cytometry. (PDF)

S3 Table Primary antibodies used for western blotting. (PDF)

S4 Table Secondary antibodies used for western blotting. (PDF)

S5 Table Primers used for qPCR. (PDF)

S1 Supplemental File RNAsequencing expression analysis of WT and Gli2/Gli3 KO fibroblasts. (XLSX)

S2 Supplemental File All numerical data for quantitation figures. (XLSX)
