# Supplementary figures and images for: Combinatorial Gli activity directs immune infiltration and tumor growth in pancreatic cancer
Source: PLoS Genet. 2022 Jul 22;18(7):e1010315. doi: 10.1371/journal.pgen.1010315 (PMC9348714; doi:10.1371/journal.pgen.1010315)

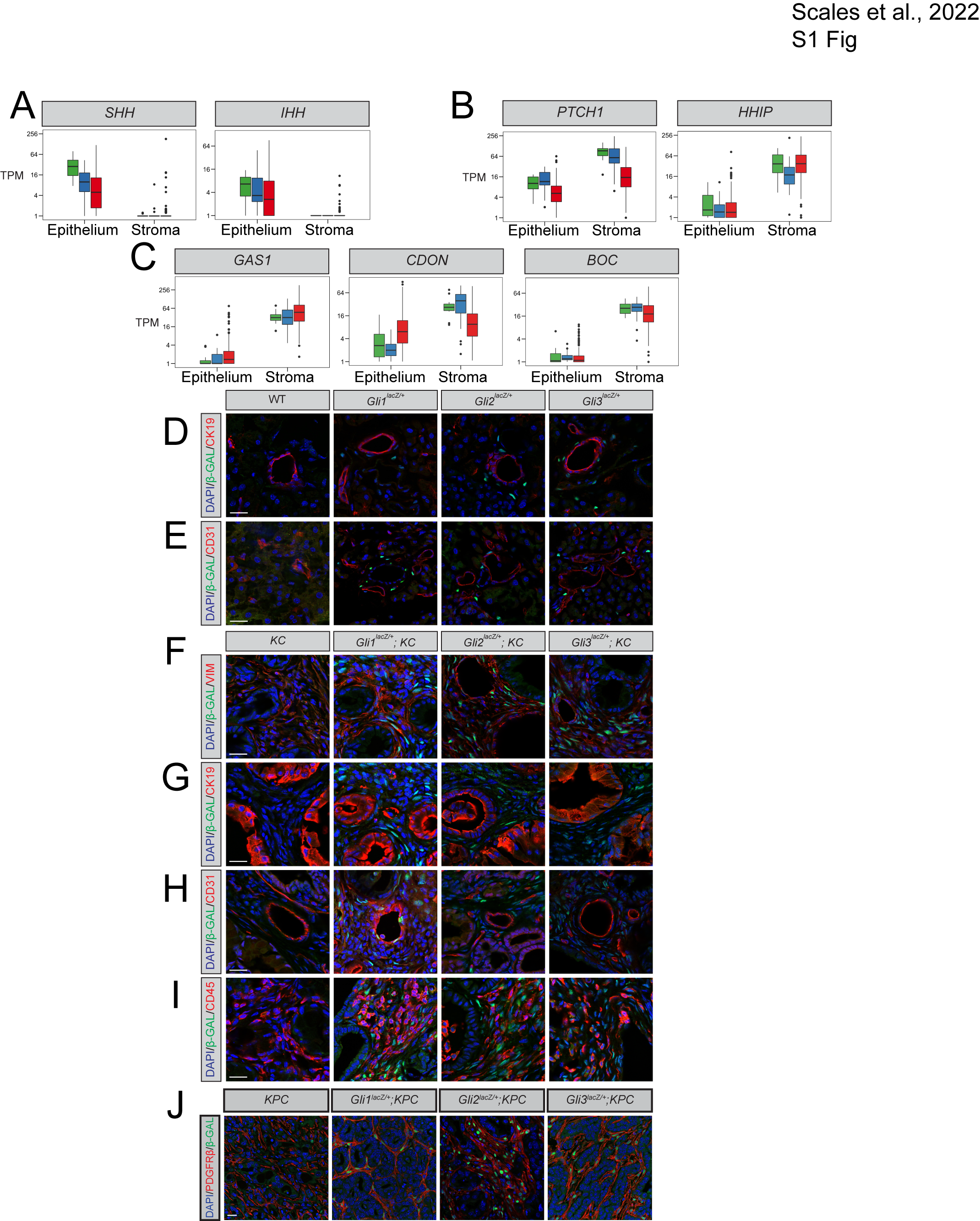

Supplement: S1 Fig — (A-C) Epithelial vs. Stromal expression of HH ligands (A) and receptors (B-C) in human IPMN (Green, n = 19 Epithelial samples, n = 12 Stromal Samples), PanIN (Blue, n = 26 Epithelial samples, n = 23 Stromal Samples), and PDA (Red, n = 197 Epithelial samples, n = 124 Stromal Samples) tissue, as determined by laser capture microdissection-RNA sequencing (40). (D-J) Immunofluorescent antibody analysis of healthy (D-E), PanIN (F-I), and tumor-bearing (J) Gli-lacZ reporter mice (n ≥ 3 for all genotypes). Antibodies detect β-Galactosidase (β-GAL, green), fibroblasts (VIM or PDGFβ, Red,F, J), ductal cells/PanIN (CK19, Red, D, G), blood vessels (CD31, Red, E, H), and immune cells (CD45, Red, I). DAPI staining in blue. Scale bar = 20μm. (TIF) [file pgen.1010315.s001.tif]

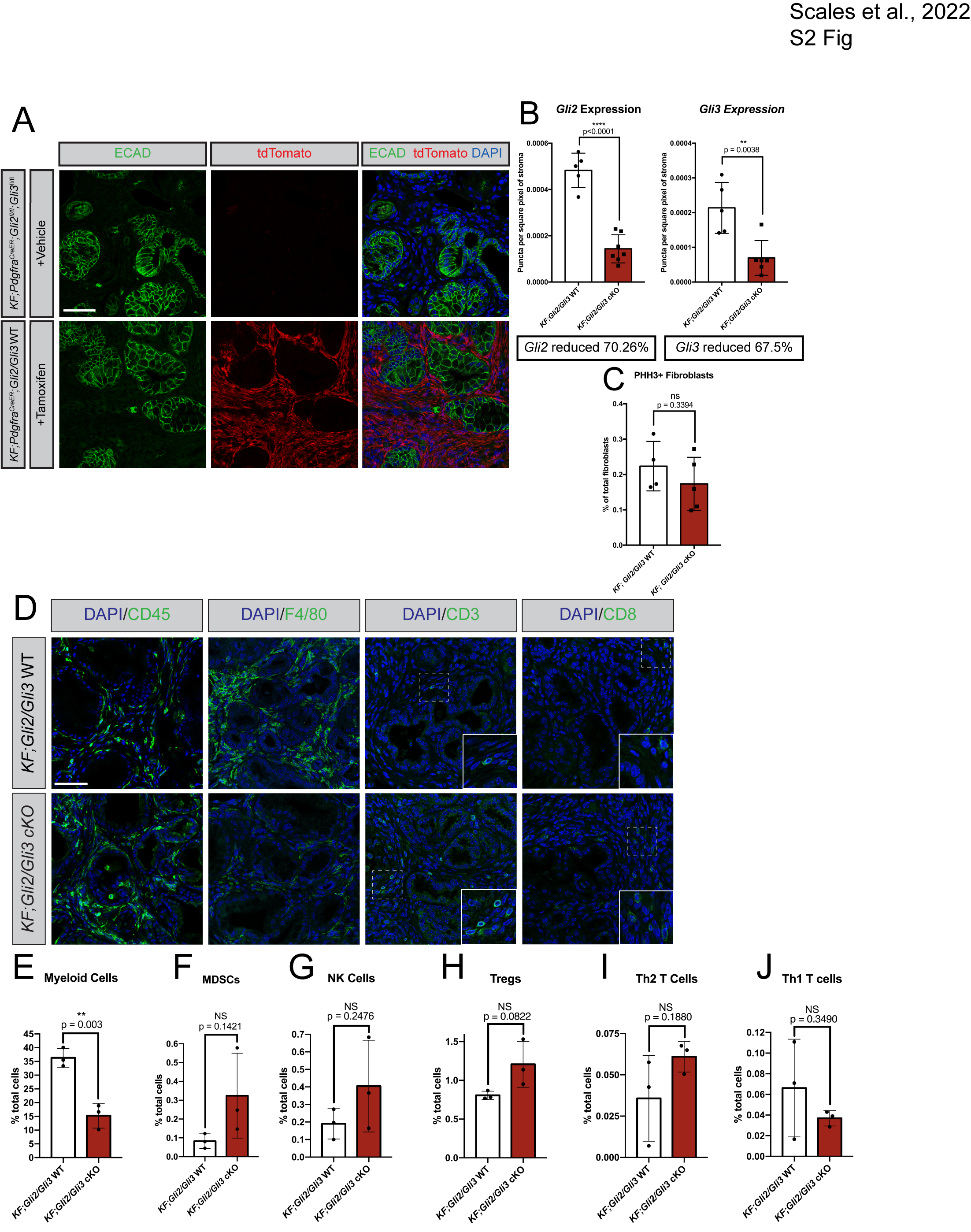

Supplement: S2 Fig — A) Immunofluorescent antibody detection of a tdTomato reporter (Red) and ECAD (Green) in KF;PdgrαCreER/+;LSL-tdTomato/+ mice treated with either tamoxifen (Bottom, n = 4) or vehicle (Top, n = 2). B) Efficiency of Gli2 (Left) and Gli3 (Right) deletion in KF;Gli2/Gli3 cKO mice, as determined by RNAscope. Puncta of Gli expression were counted and normalized to stromal area in each image. N ≥ 5 for each genotype. C) Proliferation (PHH3+) analysis of fibroblasts (PDGFRβ+) in KF;Gli2/Gli3 cKO and KF;Gli2/Gli3 WT mice (n ≥ 4 for each genotype). For (B) and (C), each point represents the average value for an animal, calculated from four independent fields of view. D) Immunofluorescent antibody detection of total immune cells (CD45), macrophages (F4/80), total T cells (CD3), and CD8+ T cells (CD8) in KF;Gli2/Gli3 WT (Top) and KF;Gli2/Gli3 cKO (Bottom) mice. DAPI staining in blue. Scale bar for all images = 50μm. E-J) Flow cytometry analysis of myeloid cells (E), MDSCs (F), NK cells (G), regulatory T cells (H), T helper 2 cells (IL4+) (I), and T helper 1 cells (IFNγ+) (J) in KF;Gli2/Gli3 WT and KF;Gli2/Gli3 cKO mice. For all immune analyses, n ≥ 3 for each genotype. For all quantitation, p-values were determined by un-paired t-test. (TIF) [file pgen.1010315.s002.tif]

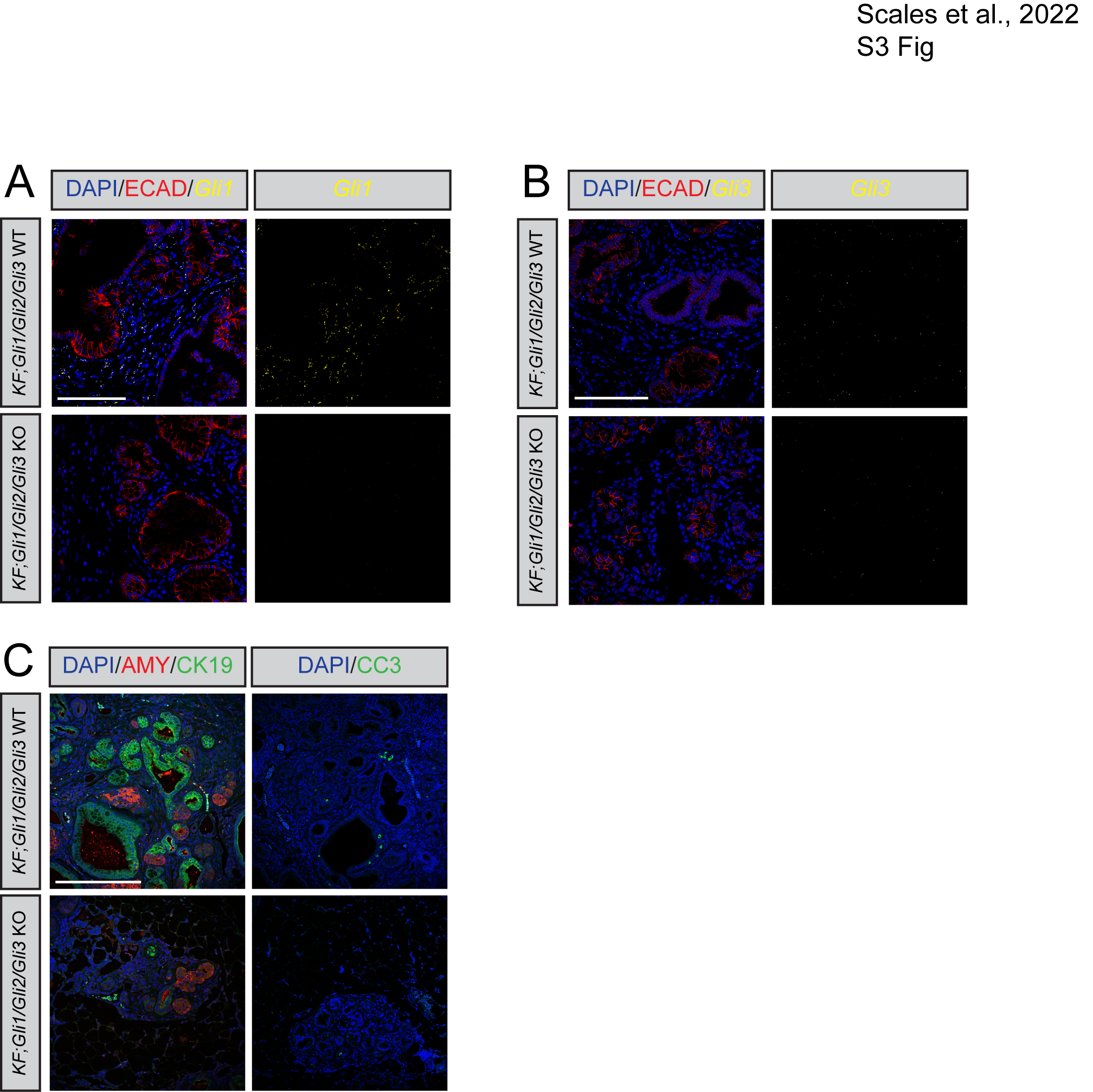

Supplement: S3 Fig — A-B) RNAscope analysis of Gli1 (A, Yellow) and Gli3 (B, Yellow) expression in KF;Gli1/Gli2/Gli3 WT (Top) and KF;Gli1/Gli2/Gli3 KO (Bottom) mice. E-Cadherin (ECAD) expressing epithelial cells in red. C) Immunofluorescent antibody detection of acinar cells (AMY, Red), PanIN lesions (CK19, Green), and cell death (CC3, Green) in KF;Gli1/Gli2/Gli3 WT (Top) and KF;Gli1/Gli2/Gli3 KO (Bottom) mice. DAPI staining in blue. Scale bar = 100 μm. N ≥ 3 for all genotypes. (TIF) [file pgen.1010315.s003.tif]

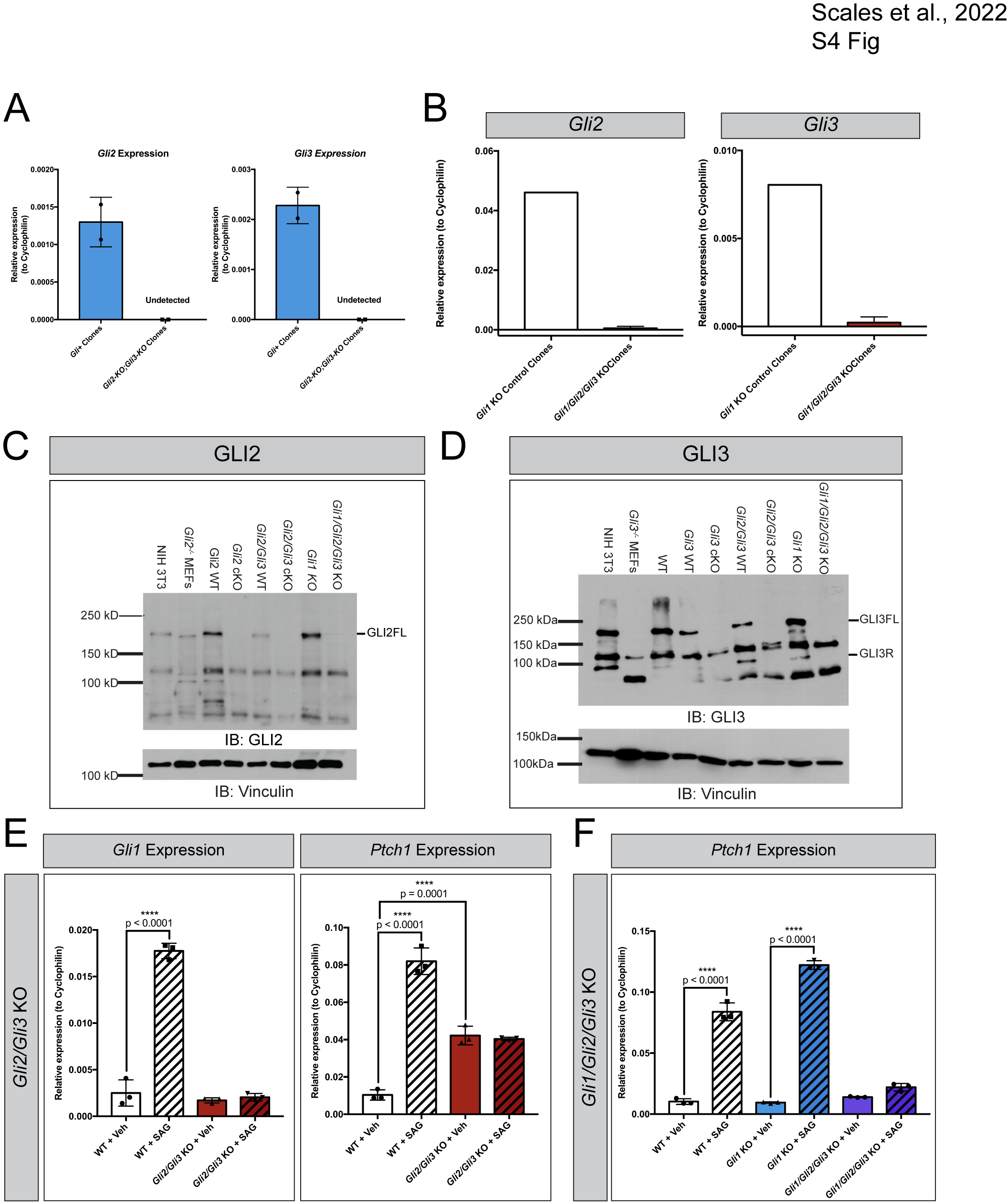

Supplement: S4 Fig — lines A-B) qPCR analysis of Gli2 and Gli3 expression in WT (A, blue bars) Gli2/Gli3 KO (A, red bars), Gli1 KO (B, white bars) and Gli1/Gli2/Gli3 KO (B, red bars) pancreatic fibroblast lines. C-D) Western Blot analysis for GLI2 (C) and GLI3 (D) in Gli WT and Gli KO pancreatic fibroblast lines. Vinculin was used as a loading control. E-F) qPCR analysis for HH target genes Gli1 and Ptch1 in Gli2/Gli3 KO (E) and Gli1/Gli2/Gli3 KO (F) pancreatic fibroblasts following treatment with vehicle or SAG (600nM). P-values determined by ordinary one-way ANOVA with Tukey’s multiple comparison test. (TIF) [file pgen.1010315.s004.tif]

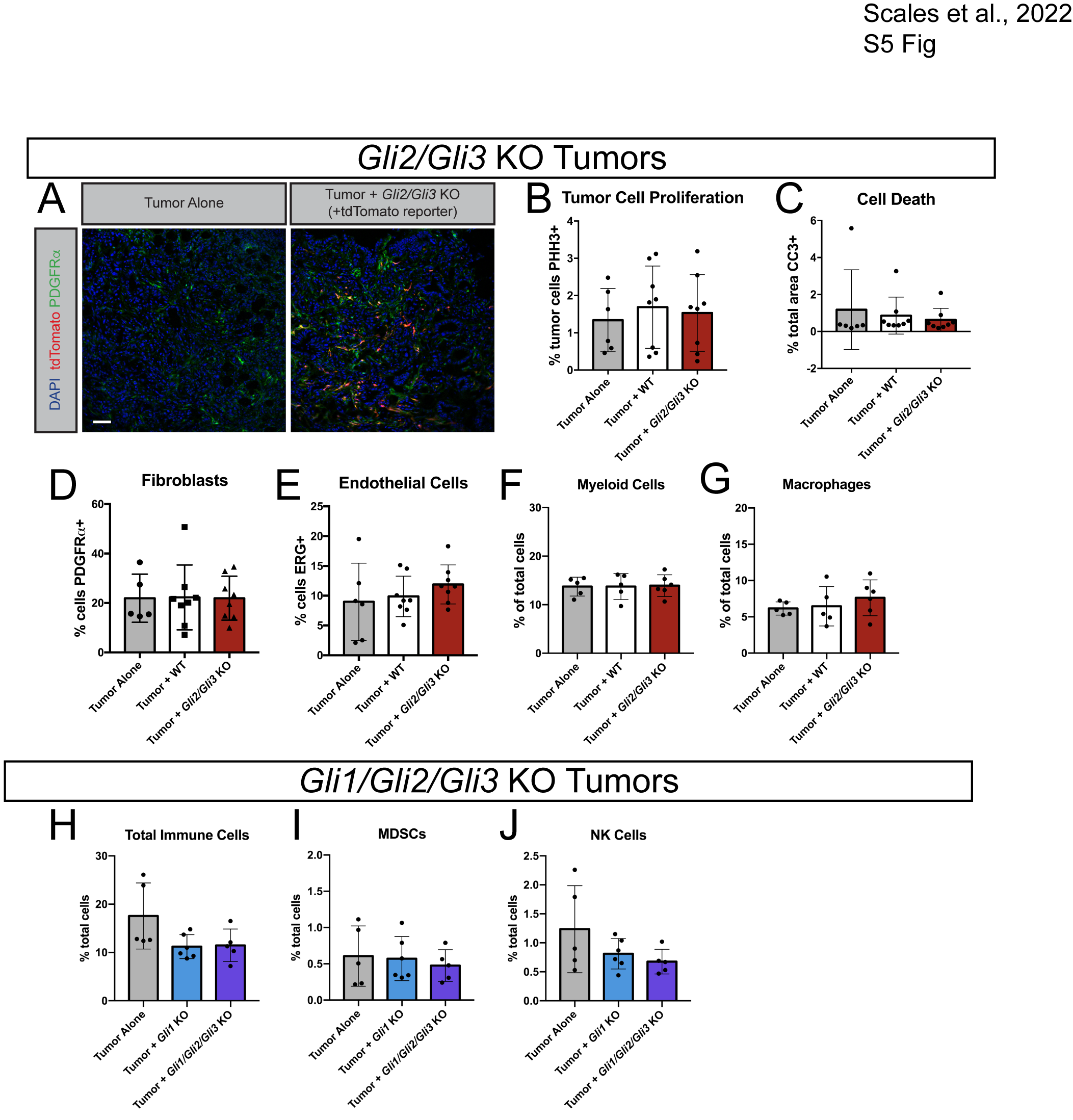

Supplement: S5 Fig — (A) Immunofluorescent antibody detection of a reporter allele (tdTomato, Red) expressed by Gli2/Gli3 KO pancreatic fibroblasts. Additional antibodies detect fibroblasts (PDGFRα, Green). DAPI staining in blue. Scale bar = 50 μm. (B-E) Quantitation of tumor cell proliferation (B), cell death (C), fibroblast number (D), and endothelial cell number (E) across experimental conditions. (F-G) Flow cytometry analysis of myeloid cells (F) and macrophages (G) from Gli2/Gli3 KO subcutaneous tumors. (H-J) Flow cytometry analysis of total immune cells (H), MDSCs (I), and NK cells (J) from Gli1/Gli2/Gli3 KO subcutaneous tumors. For all analyses, n ≥ 5 tumors for each experimental condition. Significance was determined by ordinary one-way ANOVA with Tukey’s multiple comparisons test. (TIF) [file pgen.1010315.s005.tif]

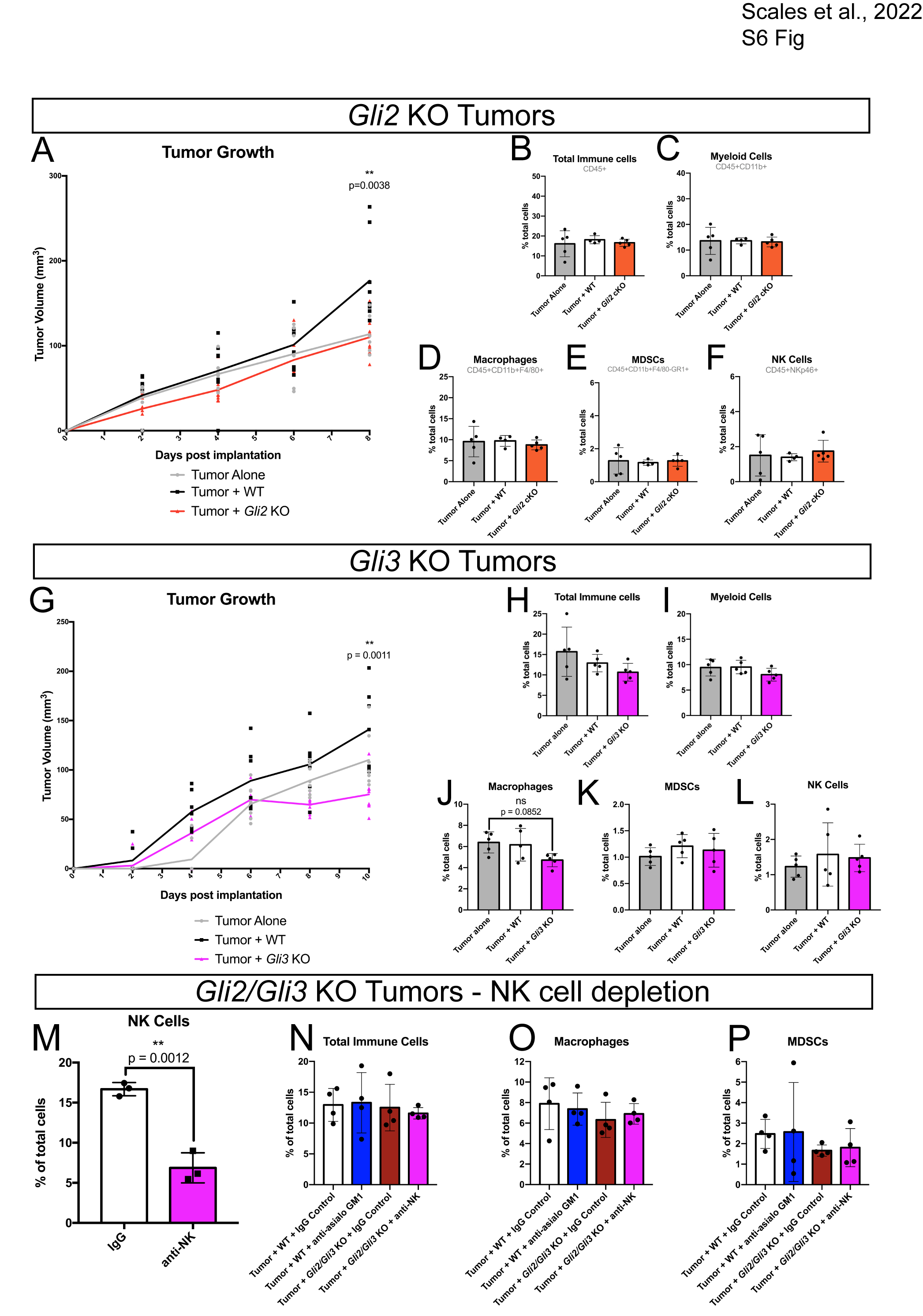

Supplement: S6 Fig — (A-L) Analysis of tumor implantation experiments incorporating Gli2 KO (A-F), Gli3 KO (G-L) pancreatic fibroblasts. (A, G) Tumor volume (mm3) over time for Gli2 KO (A) and Gli3 KO (G) tumors. The displayed p-value compares Gli KO fibroblasts to their corresponding parental line control. (B-F, H-L) Flow cytometry analysis of total immune cells (B, H), myeloid cells (C, I), macrophages (D, J), MDSCs (E, K), and NK cells (F, L) from subcutaneous tumors. (M-P) Further analysis of NK-cell depletion experiments. M) Validation of NK cell depletion in anti-NK (anti-asialo GM1)-treated mice compared to IgG control mice. P-value determined by unpaired t-test. N-P) Flow cytometry analysis of total immune cells (N), macrophages (O), and MDSCs (P). For all flow cytometry data, values displayed as a percentage of total cells. For all analyses, n ≥ 3 samples for each experimental condition. For all analyses (except M), p-values were determined by ordinary one-way ANOVA with Tukey’s multiple comparison test. (TIF) [file pgen.1010315.s006.tif]

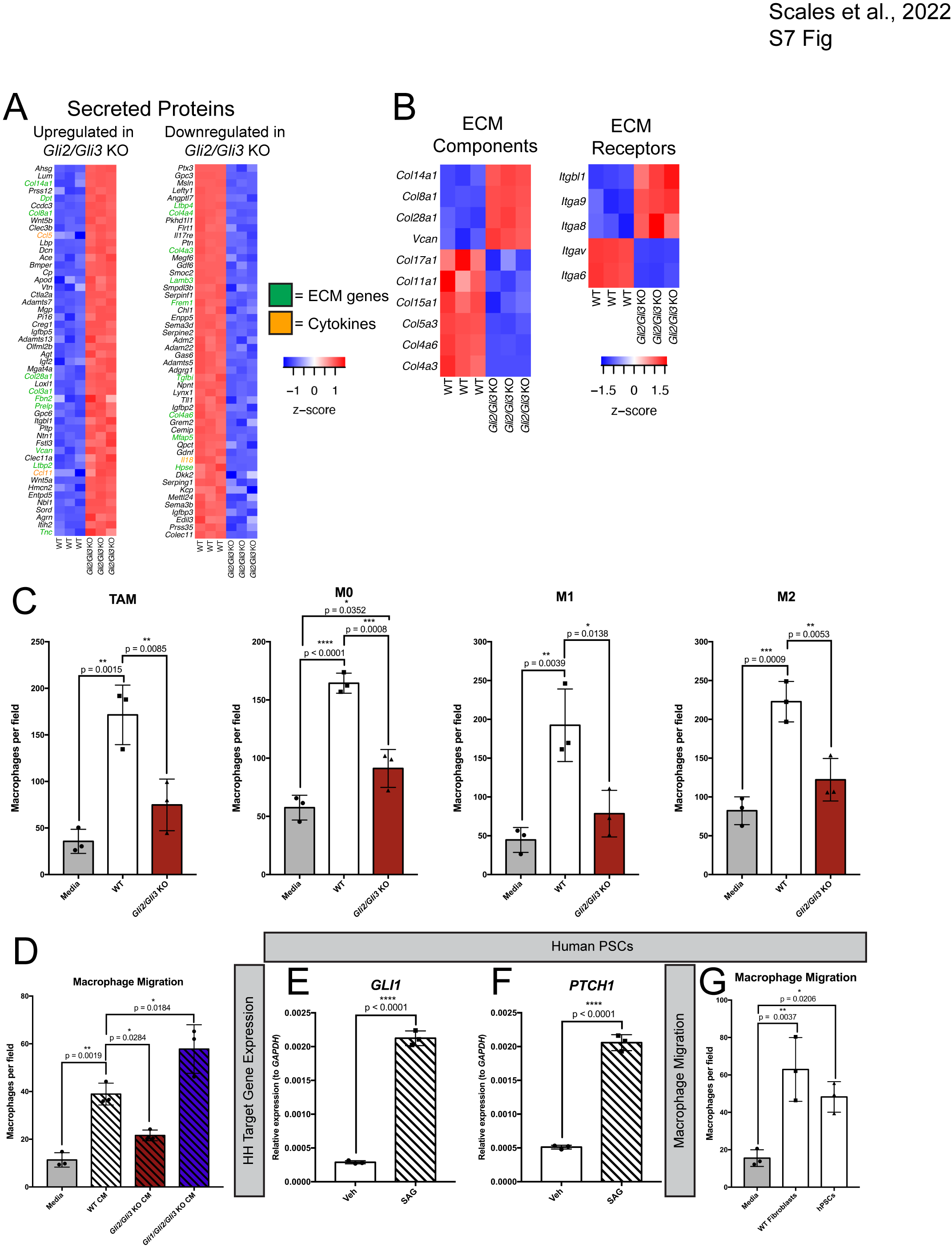

Supplement: S7 Fig — A-B) RNA sequencing analysis of Gli2/Gli3 KO pancreatic fibroblasts and Gli2/Gli3 WT pancreatic fibroblasts. A) Top upregulated (left) and downregulated (right) genes of membrane-bound and secreted proteins. Green gene names indicate ECM genes, and orange gene names indicate cytokines. B) Curated gene expression lists of ECM components (left) and ECM receptors (right). C) Migration of different macrophage phenotypes (TAM, M0, M1, M2) when co-cultured with WT or Gli2/Gli3 KO pancreatic fibroblasts. D) Macrophage migration following co-culture with pancreatic fibroblast-conditioned media. E-F) RT-qPCR analysis of HH target genes (GLI1, PTCH1) in human pancreatic stellate cells (hPSCs) following stimulation with SAG. Gene expression levels are relative to GAPDH. G) Macrophage migration following co-culture with media alone, WT mouse fibroblasts, or hPSCs. P-values for (E-F) calculated by un-paired t test. All other P-values were calculated by ordinary one-way ANOVA with Tukey’s multiple comparison test. (TIF) [file pgen.1010315.s007.tif]

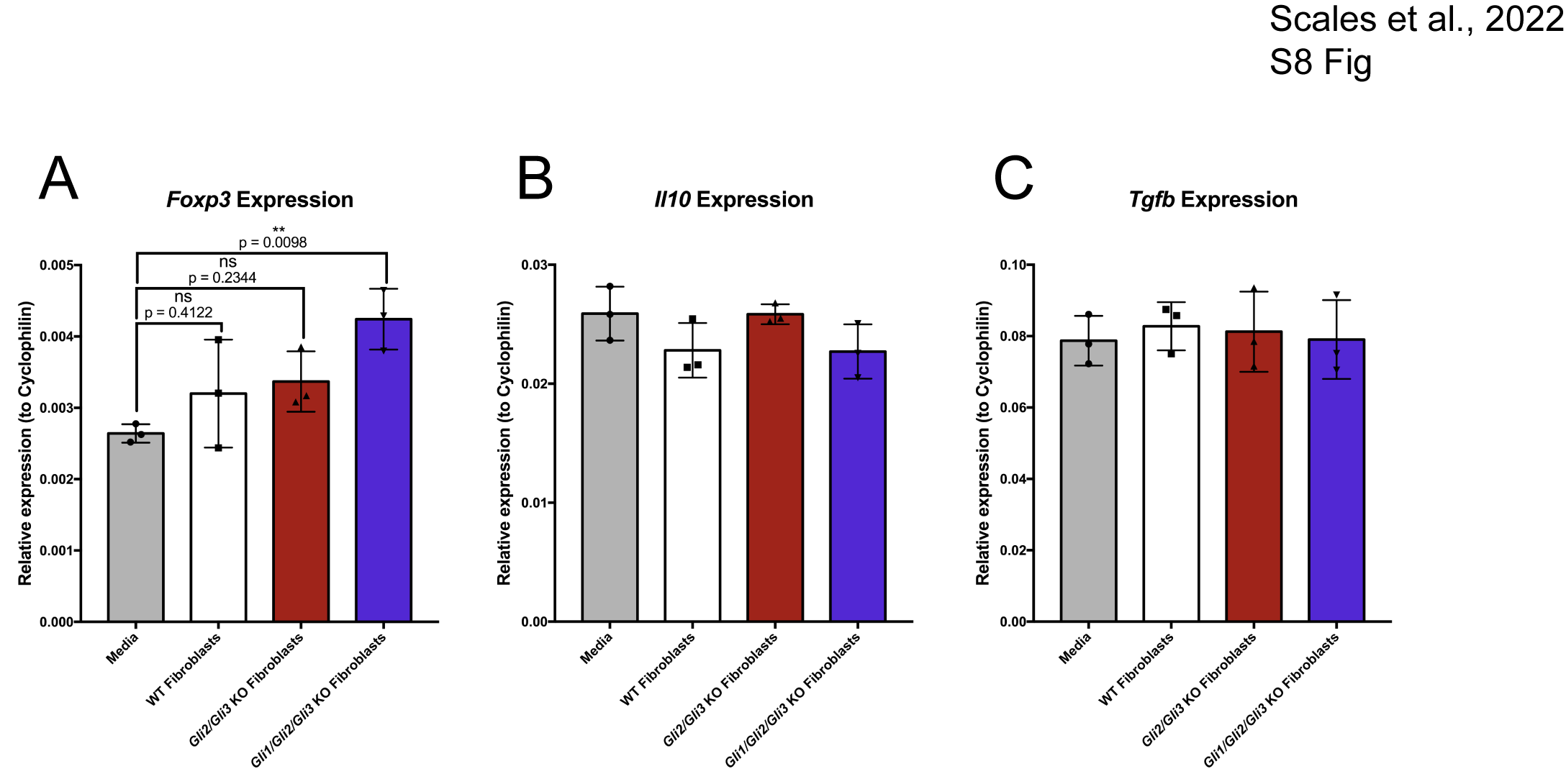

Supplement: S8 Fig — A-C) RT-qPCR analysis of T cells following transwell co-culture with pancreatic fibroblasts. Target genes include markers for Treg identity (Foxp3, A) as well as markers associated with an immunosuppressive Treg phenotype (Il10 and Tgfb, B and C, respectively). Gene expression levels are relative to Cyclophilin. P-values were determined by ordinary one-way ANOVA with Dunnett’s multiple comparison test. (TIF) [file pgen.1010315.s008.tif]
