# Supplementary material for: Combinatorial Gli activity directs immune infiltration and tumor growth in pancreatic cancer
Source: PLoS Genet. 2022 Jul 22;18(7):e1010315. doi: 10.1371/journal.pgen.1010315 (PMC9348714; doi:10.1371/journal.pgen.1010315)
Supplement: S1 Table — (PDF) [file pgen.1010315.s009.pdf]

S1 Table

Antibody	Host species	Catalog Number	Dilution
β-Gal	Chicken	Abcam ab9361	1:2500
β-Gal	Chicken	ICL Cgal-45A-Z	1:2000
Vimentin	Rabbit	Cell Signaling cs5741	1:500
CK19 (Troma-III)	Rat	DHSB AB_2133570	1:100
CD31	Rat	BD Biosciences 550274	1:500
Ecad	Mouse	BD Biosciences 610181	1:100
CD45	Rat	BD Biosciences 553076	1:100
F4/80	Rat	BMA Biomedicals T-2006	1:100
Arg1	Rabbit	Cell Signaling 93668	1:75 (TSA)
tdTomato (RFP)	Rabbit	Rockland 600-401-379	1:200
PDGFR-Beta	Rabbit	Abcam ab32570	1:200
CD3	Rabbit	Abcam ab5690	1:500
CD8	Rabbit	Cell Signaling 98941S	1:500
Alpha-Amylase	Rabbit	Sigma A8273	1:100
pHH3	Mouse	Cell Signaling 9706S	1:100
Cleaved Caspase-3 (Asp175)	Rabbit	Cell Signaling 9661S	1:200
