# Supplementary material for: Combinatorial Gli activity directs immune infiltration and tumor growth in pancreatic cancer
Source: PLoS Genet. 2022 Jul 22;18(7):e1010315. doi: 10.1371/journal.pgen.1010315 (PMC9348714; doi:10.1371/journal.pgen.1010315)
Supplement: S2 Table — (PDF) [file pgen.1010315.s010.pdf]

S2 Table

Antibodies	Host species	Catalog Number	Clone	Dilution
CD45	Rat	BD Horizon 563891	30-F11	1:100
CD11b	Rat	BD Pharmingen 557657	M1/70	1:100
F4/80	Rat	eBioscience 15-4801-82	BM8	1:100
NKp46	Rat	BD Pharmingen 560757	29A1.4	1:100
GR1	Rat	BD Pharmingen 553127	R86-8C5	1:100
CD3	Rat	BD Pharmingen 555275	17A2	1:100
CD4	Rat	BD Pharmingen 558107	RM4-5	1:100
CD8	Rat	BD Pharmingen 557654	53-6.7	1:100
Foxp3	Rat	eBioscience 53-5773-82	FJK-16s	1:100
IFN gamma	Rat	BD Pharmingen 557649	XMG1.2	1:100
IL4	Rat	BD Pharmingen 554436	11B11	1:100
