# Supplementary material for: Combinatorial Gli activity directs immune infiltration and tumor growth in pancreatic cancer
Source: PLoS Genet. 2022 Jul 22;18(7):e1010315. doi: 10.1371/journal.pgen.1010315 (PMC9348714; doi:10.1371/journal.pgen.1010315)
Supplement: S3 Table — (PDF) [file pgen.1010315.s011.pdf]

S3 Table

Antibody	Host species	Catalog Number	Dilution
GLI2	Goat IgG	R&D AF3635	1:1,000
GLI3	Goat IgG	R&D AF3690	1:1,000
Vinculin	Rabbit IgG	Cell Signaling 13901	1:1,000
