# Supplementary material for: Combinatorial Gli activity directs immune infiltration and tumor growth in pancreatic cancer
Source: PLoS Genet. 2022 Jul 22;18(7):e1010315. doi: 10.1371/journal.pgen.1010315 (PMC9348714; doi:10.1371/journal.pgen.1010315)
Supplement: S4 Table — (PDF) [file pgen.1010315.s012.pdf]

S4 Table

Secondary Antibody	Host species	Catalog Number	Dilution
anti-goat IgG	Donkey IgG	R&D HAF109	1:10,000
anti-Rabbit IgG	Donkey IgG	Jackson ImmunoResearch 711-035-152	1:5,000
