# Supplementary material for: Combinatorial Gli activity directs immune infiltration and tumor growth in pancreatic cancer
Source: PLoS Genet. 2022 Jul 22;18(7):e1010315. doi: 10.1371/journal.pgen.1010315 (PMC9348714; doi:10.1371/journal.pgen.1010315)
Supplement: S5 Table — (PDF) [file pgen.1010315.s013.pdf]

S5 Table

Target Gene	Forward Primer Sequence (5' → 3')	Reverse Primer Sequence (5' → 3')
Il6	TTCCATCCAGTTGCCTTCTTGG	TTCTCATTTCCACGATTTCCCAG
Ccl5	GCCCACGTCAAGGAGTATTT	CTTGAACCCACTTCTTCTCTGG
Il11	AGGTGGTCCTTCCCTAAAGA	GCGAGACATCAAGAGCTGTAA
Ccl7	TCAAGAGCTACAGAAGGATCACC	ATAGCCTCCTCGACCCACTT
Gli1 (mouse)	GTGCACGTTTGAAGGCTGTC	GAGTGGGTCCGATTCTGGTG
Gli2	CCTTCACCCACCTTCTTGG	CTTGTTCTGGTTGGCATCATTT
Gli3	CACATGCATCAACAGATCCTAAGC	AGGGATAGGTCTCTGTGTTGGA AAT
Ptch1 (mouse)	GAAGCCACAGAAAACCCTGTC	GCCGCAAGCCTTCTCTAGG
GLI1 (human)	CCAACTCCACAGGCATACAGGAT	CACAGATTCAGGCTCACGCTTC
PTCH1 (human)	GGGTGGCACAGTCAAGAACAG	CGTACATTTGCTTGGGAGTCATT
Il10	GCTATGCTGCCTGCTCTTACT	CCTGCTGATCCTCATGCCA
Tgfb	TGACGTCACTGGAGTTGTACGG	GGTTCATGTCATGGATGGTGC
